# Intermittent preventive treatment for malaria in pregnancy and infant growth: a mediation analysis of a randomised trial

**DOI:** 10.1016/j.ebiom.2024.105397

**Published:** 2024-10-16

**Authors:** Yanwei Tong, Kalani Ratnasiri, Suhi Hanif, Anna T. Nguyen, Michelle E. Roh, Grant Dorsey, Abel Kakuru, Prasanna Jagannathan, Jade Benjamin-Chung

**Affiliations:** a Department of Statistics, Stanford University, Stanford, United States; b Department of Epidemiology and Population Health, Stanford University, Stanford, United States; c Department of Microbiology and Immunology, Stanford University, Stanford, United States; d Institute for Global Health Sciences, University of California San Francisco, San Francisco, United States; e Department of Epidemiology and Biostatistics, University of California San Francisco, San Francisco, United States; f Department of Medicine, Division of HIV, ID, and Global Medicine, University of California San Francisco, San Francisco, United States; g Infectious Diseases Research Collaboration, Kampala, Uganda; h Department of Medicine, Stanford University, Stanford, United States; i Chan Zuckerberg Biohub, San Francisco, United States

**Keywords:** Malaria, Intermittent preventive treatment, Child growth, Mediation

## Abstract

**Background:**

Intermittent preventive treatment for malaria in pregnancy (IPTp) can improve birth outcomes, but whether it confers benefits to postnatal growth is unclear. We investigated the effect of IPTp on infant growth in Uganda and its pathways of effects using causal mediation analyses.

**Methods:**

We analysed data from 633 infants born to mothers enrolled in a randomised trial of monthly IPTp with dihydroartemisinin-piperaquine (DP) vs. sulfadoxine-pyrimethamine (SP) (NCT02793622). Weight and length were measured from 0 to 12 months of age. Using generalised linear models, we estimated effects of DP vs. SP on gravidity-stratified mean length-for-age (LAZ) and weight-for-length Z-scores (WLZ). We investigated mediation by placental malaria, gestational weight change, maternal anaemia, maternal inflammation-related proteins, preterm birth, birth length, and birth weight. Mediation models adjusted for infant sex, gravidity, gestational age at enrolment, maternal age, maternal parasitaemia at enrolment, education, and wealth.

**Findings:**

SP increased mean LAZ by 0.18–0.28 Z from birth through age 4 months compared to DP, while DP increased mean WLZ by 0.11–0.28 Z from 2 to 8 months compared to SP among infants of multigravidae; at these ages, confidence intervals for mean differences excluded 0. We did not observe differences among primigravida. Mediators of SP included birth weight, birth length, maternal stem cell factor, and DNER. Mediators of DP included placental malaria and birth length, maternal IL-18, CDCP1, and CD6 at delivery.

**Interpretation:**

In high malaria transmission settings, this exploratory study suggests different IPTp regimens may influence infant growth among multigravidae, potentially through distinct pathways, in the exclusive breastfeeding period, when few other interventions are available.

**Funding:**

10.13039/100014496Stanford Center for Innovation in Global Health, 10.13039/100009633Eunice Kennedy Shriver National Institute of Child Health and Human Development, 10.13039/100000865Bill & Melinda Gates Foundation.

**Research in context**

**Evidence before this study**

We searched PubMed for original articles using the search terms “intermittent preventive treatment” AND “sulfadoxine-pyrimethamine” AND “dihydroartemisinin-piperaquine” AND “malaria in pregnancy” AND “infant growth.” No language or time restrictions were used in this search. We did not retrieve any prior studies that examined the effects of SP vs. DP administered during pregnancy on infant growth trajectories after birth. Intermittent Preventive Treatment in Pregnancy (IPTp) with sulfadoxine-pyrimethamine (SP) is recommended by the WHO for regions with moderate-to-high malaria transmission. While SP is effective in reducing neonatal mortality and low birth weight, its efficacy has diminished in some areas of sub-Saharan Africa due to widespread parasite resistance to SP. Although IPTp with dihydroartemisinin-piperaquine (IPTp-DP) has demonstrated superior efficacy in reducing malaria in pregnancy, its impact on birth outcomes has not substantially surpassed that of SP. Prior studies also found that different IPTp regimens worked through different pathways, with DP influencing birth outcomes by reducing placental malaria and SP influencing them through non-malarial pathways such as maternal weight gain. The ultimate goal of IPTp extends beyond enhancing birth outcomes to include benefits during infancy and later stages. Yet, the effects of SP vs. DP in relation to infant growth post-birth and the underlying mechanisms remain unknown. Therefore, here, we re-analysed data from of a randomised trial in Uganda to explore the impacts of these two IPTp regimens on infant growth and to understand potential mechanisms underlying its impacts on infant growth.

**Added value of this study**

This study quantified how IPTp with SP compared to DP influenced infants’ growth trajectories, both ponderal and linear, during the first year of life. We found that SP improved linear growth of infants up to age 4 months compared to DP, and DP improved ponderal growth of infants from 2 to 8 months compared to SP among babies who were born to multigravidae. In addition, we identified birth size, placental malaria, and certain markers of maternal inflammation measured at delivery using the Olink Target 96 inflammation panel as pathways through which IPTp influenced infant growth. Our approach provides new insights into effects of IPTp beyond birth and the mechanisms by which IPTp impacts infant growth.

**Implications of all the available evidence**

Our study provides evidence that different IPTp regimens can influence infant postnatal growth through distinct pathways. Our findings highlight the potential of combined SP and DP IPTp regimens and bolster the evidence base for continued delivery of IPTp to improve maternal and child health outcomes, particularly in malaria-endemic regions.

## Introduction

Child growth faltering is associated with a large burden of disease, including increased risk of death and infections in childhood and lower productivity in adulthood.^1^, ^2^, ^3^, ^4^ Children in low-income and middle-income countries often experience growth faltering before age 6 months, when complementary feeding and most child nutrition interventions are initiated.^2^^,^^5^^,^^6^ In malaria-endemic settings, prenatal malaria infection may be an important contributor to growth failure because of its effects on inflammation, anaemia, and intrauterine growth restriction,^7^, ^8^, ^9^ which are linked to low birth weight, premature birth, stillbirth, and foetal death.^10^, ^11^, ^12^

In moderate-to-high *Plasmodium falciparum* (*Pf*) malaria transmission settings, the World Health Organization recommends intermittent preventive treatment of malaria in pregnancy (IPTp) with sulfadoxine-pyrimethamine (IPTp-SP).^13^ However, in Eastern and Southern Africa, the antimalarial efficacy of SP has waned due to increasing parasite resistance to SP.^14^ Three trials in areas of high SP resistance of Kenya and Uganda found that IPTp with dihydroartemisinin-piperaquine (IPTp-DP) reduced the risk of malaria during pregnancy, but were not associated with better birth outcomes relative to IPTp-SP.^15^, ^16^, ^17^ Ideally, IPTp would not only improve outcomes at birth but also in infancy and beyond. Yet no prior studies have assessed effects of IPTp on child growth.

SP and DP likely influence child growth through distinct pathways given DP’s higher efficacy against malaria^15^, ^16^, ^17^ and SP’s antibiotic properties.^18^ A prior study reported that effects of IPTp on birth weight were mediated by placental malaria for DP^19^; given that maternal malaria infection is associated with impaired child height and weight gain in infancy, DP’s benefits may extend into childhood.^20^, ^21^, ^22^, ^23^ Another study found that SP’s effect on birth weight was mediated by gestational weight gain,^24^ and maternal anthropometry is strongly associated with child growth.^2^ Maternal inflammation may be another important pathway: sequestration of *Pf* parasites in the placenta can result in inflammation, dysregulated development, and impaired nutrient transport in the placenta, which can negatively impact foetal development^25^ and child growth.^26^, ^27^, ^28^, ^29^, ^30^

Using data from a randomised trial of Ugandan mother-infant dyads,^17^ our objective was to assess whether IPTp-DP improved infant growth from birth to 12 months compared to IPTp-SP. An additional objective was to investigate whether maternal inflammation, anaemia, preterm birth, gestational weight gain, and birth weight and length mediated the effects of IPTp on child growth from birth to 12 months.

## Methods

### Study population

This is a secondary, pre-specified, exploratory analysis of data collected in a double-blind randomised phase III trial in Busia district, a high malaria transmission area of southeastern Uganda (NCT02793622).^17^ Genotyping of samples collected at enrolment from women from the original trial showed the prevalence of pfdhps 540E and 581G mutations (known marker of *Pf* resistance to SP) were 98% and 3% respectively.^31^ Between September 6, 2016, and May 29, 2017, the study enrolled 782 women, without HIV-infections, at least 16 years of age, and with a viable pregnancy between 12 and 20 weeks of gestation confirmed by ultrasound. The study followed their live births for 12 months from April 1, 2017 to October 31, 2018. Eligible women with a history of serious adverse events to SP or DP, early or active labour, chronic medical conditions or active medical problems requiring inpatient evaluation, or previous antimalarial therapy during the pregnancy were not enrolled in the study. Women were randomised at a 1:1 ratio to receive monthly IPTp with SP or monthly DP starting at 16 or 20 weeks of gestation. Both pharmacists administering medications and study participants were masked. We excluded multiple births (e.g., twins), spontaneous abortions, and stillbirths. The final analysis dataset included data from 633 children in the birth cohort.

### Follow-up measurements

Women were scheduled for routine visits every 4 weeks. During each visit, blood was collected to detect the presence of malaria parasites by microscopy or quantitative PCR (qPCR). Women also underwent routine laboratory testing every 8 weeks. Children born to the study participants were scheduled for visits to the clinic at 1, 4, 6, and 8 weeks of age and then every 4 weeks until they reached 52 weeks of age. Women were encouraged to visit the study clinic for all medical care for themselves or their children; the clinic was open daily, and participants received a refund for transportation costs.

Study participants, both mothers and their children, had a standardised history and physical exam taken during clinic visits which included temperature, pulse, and blood pressure measurements. Those who were febrile (tympanic temperature > 38.0 °C) or reported a fever in the past 24 h and had a positive blood smear were treated for malaria with artemether-lumefantrine for uncomplicated malaria and intravenous artesunate for complicated malaria in accordance with national treatment guidelines.

### Intervention

DP was given as 3 tablets taken once a day for 3 consecutive days every month (40 mg dihydroartemisinin and 320 mg piperaquine; Duo-Cotecxin, Holley-Cotec, Beijing). SP was given as a single dose consisting of 3 tablets every month (500 mg of sulfadoxine and 25 mg of pyrimethamine; Kamsidar, Kampala Pharmaceutical Industries, Uganda). To ensure participant blinding, participants in the DP arm received SP placebos, and participants in the SP arm received DP placebos each month. Study staff directly observed administration of the first dose of each intervention. Subsequent doses were self-administered at home, and adherence for those doses was assessed by self-report.

### Mediators

Mediators included gestational weight change, prenatal anaemia, placental malaria, preterm birth, low birth weight, and maternal inflammation. Since pre-pregnancy weight was not available, instead of measuring gestational weight gain, we calculated the change in gestational weight from 20 to 36 weeks gestation. We used weight at 36 weeks instead of delivery to increase consistency between women since the majority of women delivered after 36 weeks. Staff measured maternal haemoglobin at 28 weeks’ gestation and classified mothers as anaemic if blood haemoglobin was < 11 g/dL. Placental malaria was defined as any evidence of parasites or pigment detected in the placenta by histopathology or placental blood by microscopy, qPCR, or loop-mediated isothermal amplification (LAMP). In the analysis, we considered women who had placental malaria under either method to be positive. This captures infections detectable through microscopy and submicroscopic infections that may be missed by traditional methods but are still clinically relevant. Among 633 women included in the analyses, 281 were tested positive by histology, 75 were tested positive by LAMP, and 61 of them tested positive for both methods. We classified low birth weight as birth weight ≤2500 g and preterm birth as deliveries prior to 37 weeks’ gestation.

Using maternal plasma samples collected at delivery in a random, arm-stratified subsample of 264 mothers, we measured inflammation-related proteins using the Olink Target 96 inflammation panel (Supplementary Table S1). This high-multiplex immunoassay panel identifies 92 proteins associated with immune response. Inflammation-related protein data were log2 transformed and normalised to a Normalised Protein eXpression (NPX) relative quantification unit such that a one-unit change in NPX is equivalent to a two-fold increase in protein concentration. Twenty-five proteins were excluded from analysis because they failed to quantify in >50% of samples or the NPX value was below its limit of detection in >50% of samples (Supplementary Table S2).

### Outcomes

Infant linear growth outcomes included stunting and length-for-age Z-scores, and ponderal growth outcomes included wasting and weight-for-length Z-scores. Study staff measured length and weight in infants each month from birth to 12 months. We calculated Z-scores using the World Health Organization (WHO) Child Growth Reference Standard.^32^ We calculated mean length-for-age (LAZ), weight-for-length (WLZ), and weight-for-age (WAZ) Z-scores in intervals of 3 months. In a sensitivity analysis, we corrected Z-scores for gestational age: for infants with gestational age < 37 weeks, we calculated age as the postnatal age subtracted by difference between gestational age at birth and 280 days.

We defined stunting as LAZ < 2 standard deviations (SD) below the median of the WHO standard and wasting as WLZ < 2 SDs below the median. Severe stunting was defined as LAZ < −3, and severe wasting was defined as WLZ < −3. For binary outcomes, we calculated incidence in quarterly intervals as the number of children who became stunted or wasted during the interval divided by the number who were not previously stunted or wasted at the start of the interval. We assumed children were at risk of each outcome at birth or their first measurement.

### Statistics

The analysis plan was pre-specified at https://osf.io/f8wy4/. See deviations in Supplement S1. We did not conduct a formal sample size calculation for this exploratory analysis.

To reduce the dimensionality and identify clusters of inflammation-related proteins, we used principal components analysis and established pathway analysis and term enrichment databases (Blood Transcriptional Modules, Gene Ontology, KEGG). However, we did not observe meaningful clusters, so statistical analyses used individual proteins.

**Fig. 1:**
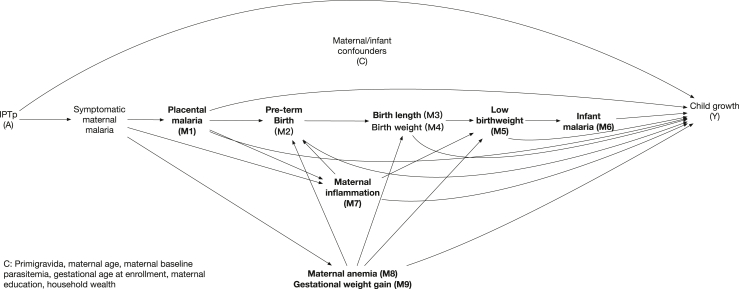
**Directed acyclic graph.** Note: all adjusted analyses in this study were controlled for the confounder set C, so for simplicity, the corresponding arrows were omitted.

To understand the mechanisms through which IPTp affects child growth faltering, we used causal mediation analyses (Fig. 1).^33^, ^34^, ^35^ We estimated total effects as the effect of IPTp on child growth faltering through all pathways, including through measured and unmeasured mediators. The total effect can be decomposed into the direct effect (i.e., “natural direct effect”) and the mediated effect (i.e., “natural indirect effect”).^36^ The direct effect measures the effect of IPTp on child growth if we disabled pathways through mediators, while the mediated effect measures the effect of IPTp on child growth that operates through mediators.

We estimated total effects of IPTp DP vs. SP using generalised linear models with a Gaussian family and identity link for continuous outcomes (Z-scores) and generalised linear models with a Poisson family and log link for dichotomous outcomes (stunting and wasting).^37^ We fit unadjusted models because characteristics were balanced at baseline between study groups.^17^

We fit models to estimate effects of IPTp (DP vs. SP) on mediators and associations between mediators and each outcome using generalised linear models as defined above. We report crude intervention-mediator effects since the intervention was randomised. For mediation analyses, mediator and outcome models adjusted for mediator-outcome confounders by choosing nodes sufficient to block backdoor pathways in Fig. 1. Potential confounders measured in the original trial included maternal gravidity, infant sex, gestational age at enrolment, maternal age, maternal parasitaemia status at enrolment, education, and household wealth. Using the mediation R package, we estimated mediation parameters and obtained 95% confidence intervals using a quasi-Bayesian Monte Carlo approach with 1000 simulations.^38^ The method used in the mediation package is “quasi-Bayesian” in that it uses Monte Carlo simulation to approximate the sampling distribution of the mediation effects, rather than specifying full prior distributions. By using normal approximation based on the parameter estimates, the method avoids the need to manually specify prior distributions while still capturing uncertainty in the estimates. We used 1000 iterations for the posterior distribution to maintain consistency and comparability with Roh et al.^19^ We compared outcome models with and without intervention-mediator interaction terms; because results were similar, we report estimates from models without interaction terms. Analyses of inflammation-related proteins were restricted to the random subsample in which we performed Olink. We corrected p-values for Olink analyses using Benjamini-Hochberg correction (false discovery rate p-value < 0.05).^39^ Other analyses considered a smaller number of mediators and were not corrected for multiple testing. We compared characteristics at baseline and at birth for infant-mother dyads with and without complete follow-up at 12 months and performed a complete case analysis.

Identification assumptions of causal mediation analyses include no unmeasured confounding of the intervention-outcome relationship, mediator-outcome relationship, or intervention-mediator relationship; temporal ordering intervention, mediators, and outcomes; and no mediator-outcome confounder that is itself affected by the intervention.^34^ The first assumption is met because intervention was randomised. We adjusted for confounders in intervention-outcome and mediator-outcome models to minimise confounding. Intervention, mediators, and outcomes were temporally sequenced.

### Ethics

This study was approved by the Institutional Review Board at Stanford University (#40857); the original trial was approved by ethics committees of Makerere University School of Biomedical Sciences (Kampala, Uganda, approval number SBS-342), the Uganda National Council for Science and Technology (Kampala, Uganda; HS 2052). All study participants provided written informed consent.

### Role of funders

The funders of the study had no role in study design, data collection, data analysis, data interpretation, or writing of the report. The corresponding author had full access to all the data in the study and had final responsibility for the decision to submit for publication.

## Results

### Characteristics of the study population

**Fig. 2:**
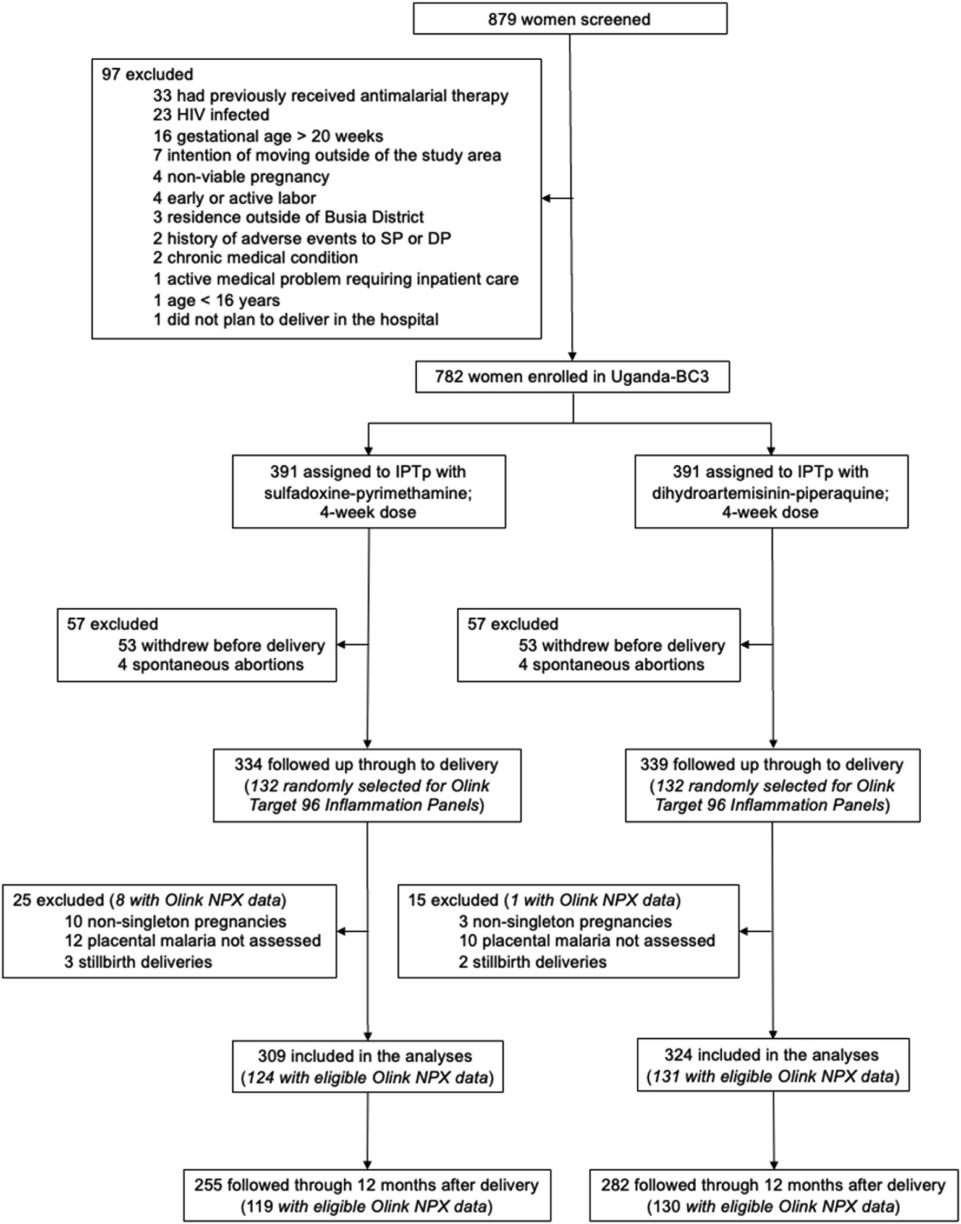
**Flowchart of participants included in this study**.

The study initially enrolled 782 women. This analysis excluded 149 individuals due to study withdrawal, spontaneous abortions, stillbirth, non-singleton birth, and missing placental malaria measurements. The analysis sample included 633 singleton mother-infant dyads (Fig. 2), 152 (24%) of which were primigravidae. Mothers’ mean age was 24 years (SD = 6) (Table 1). The average gestational weight change between week 20 and week 36 was 3.4 kg. We documented 296 cases of anaemia and 34 (5%) preterm deliveries. The prevalence of active or past placental malaria measured at delivery was 80% among primigravidae and 33% among multigravidae. At birth, the mean gestational age was 39 weeks (SD = 3). 537 mother-infant dyads were followed through 12 months after delivery. The percentage of live births with anthropometric measurements was 98% at birth, 89% at 6 months, and 78% at 12 months; follow-up was similar between arms. Comparing mother-infant dyads with and without complete follow-up through age 12 months, overall, characteristics were similar; those with incomplete follow-up were more likely to be primigravidae, have had placental malaria, or have been born with low birth weight (Supplementary Table S3).

**Table 1:** Characteristics of mother-infant dyads followed through 12 months after delivery.

	SP (n = 255)	DP (n = 282)
**Maternal characteristics at baseline**		
Mother’s age at enrolment (years)	24 (19–29)	23 (20–28)
Gestational age at enrolment (weeks)	15.9 (13.4, 17.9)	15.0 (13.4, 17.0)
Gestational age category at enrolment (weeks)		
12–16	134 (53%)	169 (60%)
>16–20	121 (47%)	113 (40%)
Gravidity		
1	58 (23%)	59 (21%)
2	55 (22%)	70 (25%)
≥3	142 (56%)	153 (54%)
Parasite prevalence by microscopy or qPCR at enrolment	212 (83%)	227 (80%)
Haemoglobin concentration at enrolment (g/dL)	11.6 (10.7–12.4)	11.4 (10.7–12.2)
**Infant characteristics at birth**		
Gestational age at delivery (weeks)	39.9 (38.9–40.6)	39.9 (38.9–40.7)
Preterm birth	12 (5%)	11 (4%)
Infant sex		
Female	128 (50%)	149 (53%)
Male	127 (50%)	133 (47%)
Low birth weight	13 (5%)	14 (5%)

Data are median (IQR) or n (%).

### Child growth faltering

At birth, median LAZ was −1.0 (IQR = 1.05) and median WLZ was 0.55 (IQR = 1.63); mean LAZ was −0.99 (95% CI -1.07, −0.91) and mean WLZ was 0.49 (95% CI 0.40, 0.59) (Supplementary Fig. S1a and b). The proportion of children experiencing incident stunting onset was 18% at birth and 23% from age 1 day to 3 months, and <10% at subsequent ages (Supplementary Fig. S1c). At each age, the incidence of severe stunting was 6% or lower, with higher incidence before age 3 months. Wasting incidence was 13% from 1 day to 3 months and was <4% at all other ages (Supplementary Fig. S1d). Severe wasting incidence was <3% at all ages.

### Effects of IPTp on child growth

**Fig. 3:**
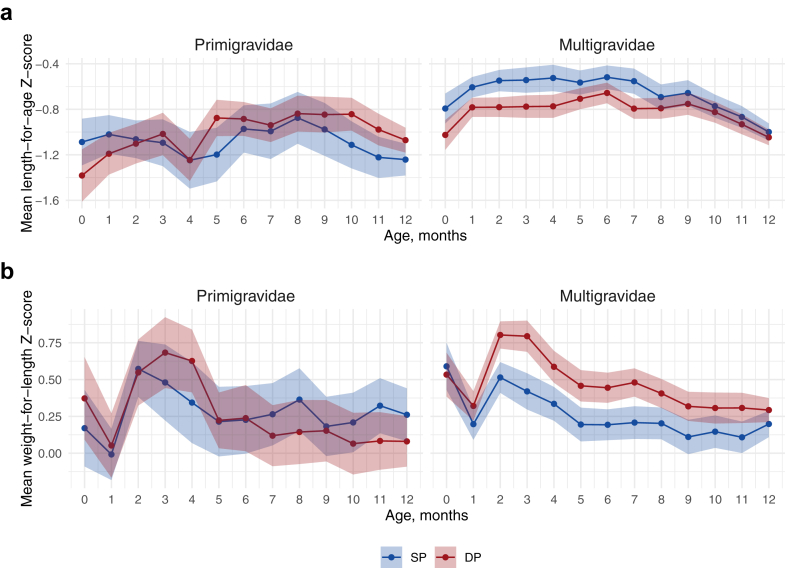
**Total effect of IPTp DP vs. SP on mean child growth Z-scores by child age and gravidity.** Includes 633 children measured from birth to twelve months. Z-scores compare children’s length and/or weight to the WHO Child Growth Standards for a given age and sex. The shaded region represents the 95% confidence interval, coloured by IPTp group (DP vs. SP).

Among all infants, mean LAZ was 0.23 (95% CI 0.07, 0.40) higher at birth and 0.17 (95% CI 0.02, 0.33) higher from 1 day to 3 months in the SP arm compared to DP (Table 2). Mean WLZ was similar at birth but 0.19 (95% CI 0.04, 0.34) higher in the DP arm compared to SP from age 1 day to 3 months. WLZ and LAZ differences between arms were smaller from ages 6–12 months. When stratifying by gravidity, we observed differences only among multigravidae: SP increased mean LAZ by 0.19–0.27 Z from birth to 4 months compared to DP, with mean LAZ around −0.55 in the SP arm and −0.75 in the DP arm at these ages (Fig. 3). Compared to SP, DP increased mean WLZ from 2 to 8 months by 0.11–0.28 Z. Gestational age correction increased LAZ by approximately 0.2 Z and WLZ by approximately 0.5 Z and did not change patterns by arm (Supplementary Fig. S2). The incidence of stunting and wasting by age was similar between study arms (Table 2).

**Table 2:** Total effect of IPTp-DP vs. IPTp-SP on infant growth.

Age category	SP		DP		Unadjusted mean difference (95% CI)
	N	Mean (95% CI)	N	Mean (95% CI)	
Length-for-age Z					
Birth	303	−0.87 (−0.98, −0.76)	319	−1.10 (−1.22, −0.99)	−0.23 (−0.40, −0.07)
1 day-3 months	296	−0.70 (−0.81, −0.59)	311	−0.87 (−0.97, −0.77)	−0.17 (−0.33, −0.02)
>3–6 months	285	−0.64 (−0.77, −0.51)	302	−0.76 (−0.86, −0.66)	−0.12 (−0.28, 0.04)
>6–9 months	275	−0.67 (−0.81, −0.54)	294	−0.77 (−0.87, −0.66)	−0.10 (−0.26, 0.07)
>9–12 months	266	−0.93 (−1.04, −0.82)	292	−0.94 (−1.04, −0.84)	−0.01 (−0.16, 0.14)
Weight-for-length Z					
Birth	294	0.48 (0.34, 0.63)	302	0.50 (0.36, 0.63)	0.01 (−0.18, 0.21)
1 day-3 months	289	0.39 (0.28, 0.50)	305	0.58 (0.47, 0.69)	0.19 (0.04, 0.34)
>3–6 months	287	0.31 (0.18, 0.43)	299	0.42 (0.3, 0.53)	0.11 (−0.06, 0.28)
>6–9 months	274	0.23 (0.10, 0.35)	294	0.31 (0.19, 0.43)	0.09 (−0.09, 0.26)
>9–12 months	266	0.18 (0.05, 0.31)	292	0.23 (0.11, 0.35)	0.05 (−0.13, 0.23)
		**Incidence (95% CI)**		**Incidence (95% CI)**	**Unadjusted incidence ratio (95% CI)**
Stunting					
Birth	303	14.5 (11.0, 18.9)	319	18.5 (14.6, 23.1)	1.27 (0.86, 1.88)
1 day-3 months	252	19.8 (15.4, 25.2)	255	26.3 (21.3, 32.0)	1.32 (0.92, 1.91)
>3–6 months	197	8.6 (5.5, 13.4)	182	10.4 (6.8, 15.7)	1.21 (0.63, 2.33)
>6–9 months	174	8.6 (5.3, 13.7)	160	8.1 (4.8, 13.4)	0.94 (0.45, 1.98)
>9–12 months	153	2.0 (0.7, 5.6)	147	4.8 (2.3, 9.5)	2.43 (0.63, 9.39)
Wasting					
Birth	294	3.1 (1.6, 5.7)	302	2.6 (1.3, 5.1)	0.87 (0.33, 2.24)
1 day-3 months	280	12.5 (9.1, 16.9)	297	11.4 (8.3, 15.6)	0.92 (0.57, 1.47)
>3–6 months	241	3.3 (1.7, 6.4)	256	4.3 (2.4, 7.5)	1.29 (0.52, 3.22)
>6–9 months	224	2.7 (1.2, 5.7)	244	2.5 (1.1, 5.3)	0.92 (0.30, 2.85)
>9–12 months	211	2.8 (1.3, 6.1)	236	2.5 (1.2, 5.4)	0.89 (0.29, 2.77)

Note: Z-scores whose absolute value greater than 6 were considered as extreme values. Such Z-scores and their corresponding binary outcomes were excluded from analyses.

### Effects of IPTp on mediators

**Fig. 4:**
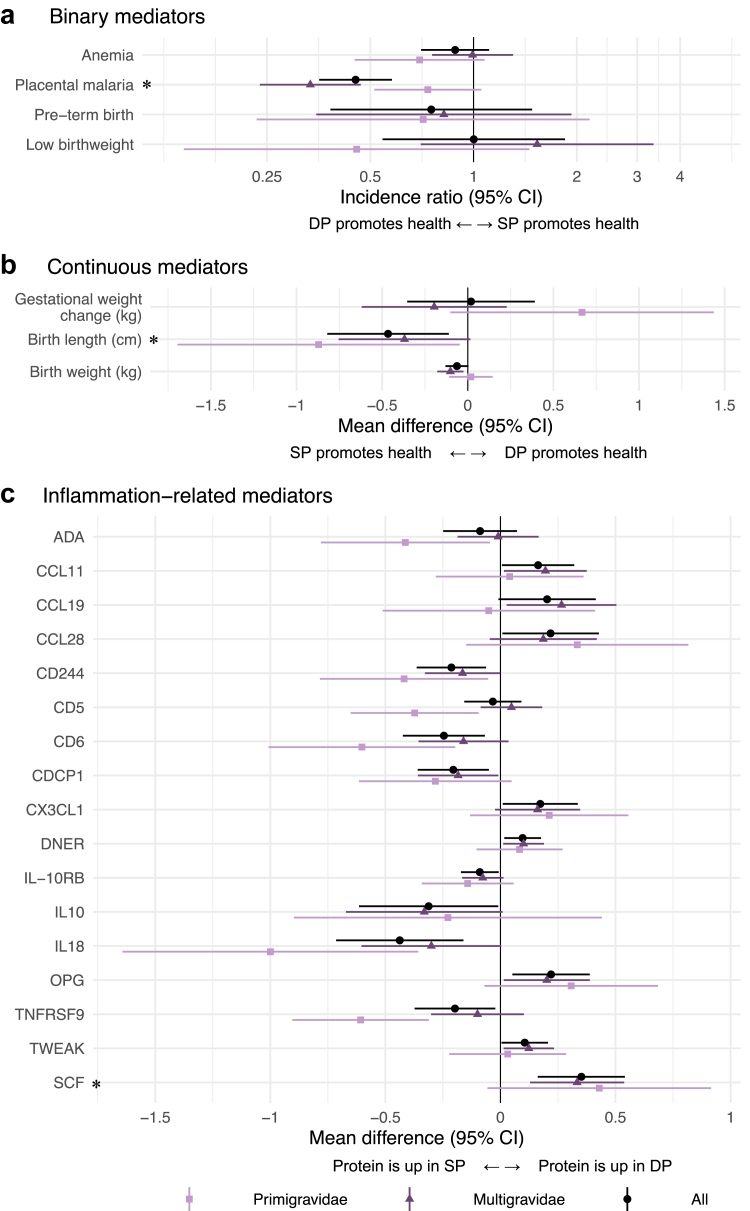
**Effect of IPTp-DP vs. IPTp-SP on potential mediators.** Each panel shows unadjusted incidence ratios and mean differences for each mediator between IPTp-DP and IPTp-SP. Panel a) includes binary mediators, panel b) includes continuous mediators, and panel c) includes continuous inflammation-related protein mediators measured in the Olink panel. Colours indicate gravidity strata. The asterisk indicates a statistically significant effect among all gravidae. In Olink inflammation analyses, the asterisk indicates statistical significance after Benjamini-Hochberg correction (false discovery rate p-value < 0.05). Panel c) only includes Olink results with statistically significant differences between arms; results for all Olink proteins are shown in Supplementary Fig S4. Panels a) includes N ∈ [619, 633] children, panel b) includes N ∈ [575, 633] children, and panel c) includes N = 255 children with data from Olink analyses. 95% CI = 95% confidence interval. ADA = Adenosine deaminase. CCL11 = Eotaxin. CCL19 = C-C motif chemokine 19. CCL28 = C-C motif chemokine 28. CD224 = Natural killer cell receptor 2B4. CD5 = T-cell surface glycoprotein CD5. CD6 = T-cell differentiation antigen CD6. CDCP1 = CUB domain-containing protein 1. CX3CL1 = Fractalkine. DNER = Delta and Notch-like epidermal growth factor-related receptor. IL-10RB = Interleukin-10 receptor subunit beta. IL10 = Interleukin-10. IL18 = Interleukin-18. OPG (TNFRSF11B)=Osteoprotegerin (Tumour necrosis factor receptor superfamily member 11B). TNFRSF9 = Tumour necrosis factor receptor superfamily member 9. TWEAK (TNFSF12) = Tumour necrosis factor (ligand) superfamily, member 12. SCF (KITLG) = Stem cell factor (Kit ligand).

SP improved mean birth length by 0.47 cm (95% CI 0.11, 0.82) and mean birth weight by 60 g (95% CI 0, 130) compared to DP (Fig. 4). For birth length, the effect was larger among primigravidae, but for birth weight, the effect was only present among multigravidae. Placental malaria was lower for DP compared to SP (incidence ratio = 0.45, 95% CI 0.35, 0.58), and the effect was stronger among multigravidae.

We identified 17 inflammation-related proteins that were either upregulated in DP relative to SP or vice versa. Certain inflammation-related proteins were upregulated in DP relative to SP at delivery, including Fractalkine (CX3CL1), Delta and Notch-like epidermal growth factor-related receptor (DNER), Osteoprotegerin (OPG), and SCF. CD244, T cell surface glycoprotein CD6 isoform (CD6), CUB domain-containing protein 1 (CDCP1), interleukin-18 (IL-18), and tumour necrosis factor receptor superfamily member 9 (TNFRSF9) were upregulated in SP compared to DP. The largest effects were on OPG, IL-18, and SCF. Compared to SP, DP upregulated SCF by 0.35 NPX (95% CI 0.16, 0.54), upregulated OPG by 0.24 NPX (95% CI 0.08, 0.41), and downregulated IL-18 by 0.48 NPX (95% CI 0.20, 0.76). These are equivalent to 70%, 48%, and −96% percent changes, respectively. After applying the Benjamini-Hochberg correction, only stem cell factor (SCF) was upregulated in DP (Fig. 4, Supplementary Fig. S3). We did not observe differences in anaemia, pre-term birth, low birth weight, gestational weight change, or other inflammation-related proteins between arms.

### Association between mediators and child growth

After adjusting for treatment arm and other potential confounders (Fig. 1), mediators strongly associated with LAZ and WLZ included pre-term birth, birth weight, and birth length (Supplementary Fig. S4). Placental malaria was associated with 0.30 (95% CI 0.03, 0.57) lower mean LAZ at birth among multigravidae, but there was no association at other ages or among primigravidae. Preterm birth was associated with higher stunting incidence at birth and higher stunting and wasting incidence from 1 day to 3 months (Supplementary Table S4). Birth length was associated with lower stunting incidence from birth through 9 months and higher wasting incidence at birth. Birth weight was associated with lower stunting and wasting incidence at certain ages. Multiple inflammation-related proteins were associated with LAZ, WLZ, stunting, and wasting (Supplementary Fig. S5 and S6). After multiple testing correction, only two protein associations with WLZ at certain ages were statistically significant (Supplementary Fig. S5).

### Mediators of the effect of IPTp on linear growth

**Fig. 5:**
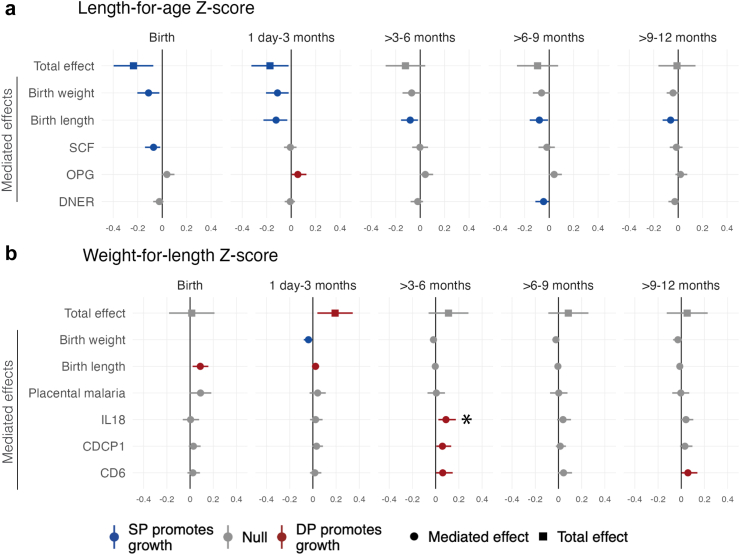
**Total effects and mediated effects on child growth Z-scores.** The total effects compare mean Z-scores between IPTp-DP and IPTp-SP using unadjusted models. The mediated effects of IPTp-DP vs. IPTp-SP on Z-scores were adjusted by infant sex, maternal age, maternal baseline parasitaemia, gestational age at enrolment, maternal education, household wealth, and gravidity. The reference group was SP. Includes all gravidae. For non-Olink mediators, the mediated effects in panel a) includes data from N = 622 children at birth, N ∈ [605, 606] from 1 day to 3 months, N ∈ [585, 587] from >3 to 6 months, N ∈ [567, 569] from >6 to 9 months, and N ∈ [557, 558] from >9 to 12 months; the mediated effects in panel b) includes data from N = 596 children at birth, N ∈ [592, 594] from 1 day to 3 months, N ∈ [584, 586] from >3 to 6 months, N ∈ [566, 568] from >6 to 9 months, and N ∈ [557, 558] from >9 to 12 months. For Olink mediators, the mediated effects in panel a) includes data from N = 255 children at birth, N = 251 from 1 day to 3 months, N = 255 from >3 to 6 months, N = 255 from >6 to 9 months, and N = 255 > 9–12 months; the mediated effects in panel b) includes data from N = 245 children at birth, N = 247 from 1 day to 3 months, N = 255 from >3 to 6 months, N = 254 from >6 to 9 months, and N = 255 from >9 to 12 months. The sample sizes for total effects can be found in Table 2. ∗Statistically significant among all gravidae after Benjamini-Hochberg correction (false discovery rate p-value < 0.05). SCF (KITLG) = Stem cell factor (Kit ligand). OPG (TNFRSF11B)=Osteoprotegerin (Tumour necrosis factor receptor superfamily member 11B). DNER = Delta and Notch-like epidermal growth factor-related receptor. IL18 = Interleukin-18. CDCP1 = CUB domain-containing protein 1. CD6 = T-cell differentiation antigen CD6.

Mediators of the effect of SP compared to DP on LAZ included birth weight and birth length and inflammation-related proteins SCF and DNER measured at delivery (Fig. 5a, Supplementary Fig. S1, Supplement S2). Birth weight and birth length were the strongest mediators. Birth weight showed mediating effects ranging from 0.04 to 0.11, with the most notable effects observed from birth through 3 months. Birth length showed mediating effects ranging from 0.06 to 0.25 across all ages, with stronger effects at younger ages. Some inflammation-related proteins showed smaller mediating effects, with heterogeneity in effects by age. Reductions in SCF at delivery mediated SP’s effects on LAZ at birth (Benjamini-Hochberg (BH) p = 0.072). Downregulation of DNER at delivery had a small mediating effects on LAZ, particularly at 6–9 months (BH p = 0.504). Compared to SP, DP increased LAZ upregulating OPG at delivery; the mediating effect was present at all ages and strongest from 1 day to 3 months (BH p = 0.396). The effect of IPTp on stunting at birth was mediated by pre-term birth, low birth weight, and SCF at delivery (BH p = 0.543) (Supplementary Fig. S8). Birth weight and birth length were also mediators of stunting from 1 day to 3 months. For linear growth outcomes, there was no evidence of mediation by placental malaria, maternal anaemia, preterm birth, gestational weight change, or other inflammation-related proteins (Supplementary Figs. S9–S11 and Table S5).

### Mediators of the effect of IPTp on ponderal growth

Mediators of the effect of DP compared to SP on WLZ included birth size up to 3 months; certain inflammation-related proteins measured in mothers at delivery were mediators for WLZ later in infancy (Fig. 5b, Supplementary Fig. S7, Supplement S2). Birth length showed mediated effects of 0.02–0.09 from birth through 3 months, reflecting lower birth lengths and greater weight gain in DP vs. SP. Relative to DP, SP increased WLZ through its effect on birth weight, with the strongest mediating effects observed from birth through 3 months. Placental malaria showed mediating effects on WLZ ranging from 0.04 to 0.09 from birth through 3 months, however, the confidence interval included the null (Supplementary Table S5). Inflammation-related proteins showed smaller, age-varying mediating effects. Relative to SP, DP improved WLZ by downregulating IL-18, CDCP1, and CD6 at delivery. Mediating effect sizes were 0.02–0.06 for DC6, 0.02–0.06 for CDCP1, and 0.01–0.09 for IL-18. After multiple testing correction, the only BH p-value < 0.05 was for IL-18 at 3–6 months. The effect of IPTp on wasting was mediated by low birth weight and birth weight up to age 3 months (Supplementary Fig. S8). There was no evidence of mediation of the effect of DP or SP on WLZ or wasting by placental malaria, maternal anaemia, preterm birth, gestational weight change, or other inflammation-related proteins (Supplementary Figs. S9, S12, and S13 and Table S5).

## Discussion

In this secondary analysis of a randomised trial of Ugandan mother-infant dyads which found no difference in birth outcomes for IPTp-DP vs. IPTp-SP,^17^ we found that IPTp-SP improved linear growth from birth to age 4 months compared to DP, and IPTp-DP improved ponderal growth from ages 2–8 months compared to SP among infants born to multigravidae. We did not observe differences between IPTp regimens on child growth in primigravidae, possibly because the adverse consequences of placental malaria are more severe in this subgroup, which may have offset the ‘non-malarial’ benefits of SP.

Our mediation analysis found that IPTp regimens influenced infant growth through distinct pathways, consistent with a prior study that focused on birth outcomes.^19^ SP increased linear and ponderal growth via increased birth size. DP increased ponderal growth through reduced inflammation and reduced placental malaria, consistent with a prior analysis,^19^ though evidence was weak for the latter. SP’s effects on linear growth and DP’s effects on ponderal growth were mediated by reductions in different inflammation-related proteins, with the strongest evidence for mediation of DP’s effect on ponderal growth by reduced IL-18. Mediation analysis can provide insights into the distinct mechanisms of IPTp regimens, even in the absence of an overall treatment difference in the original analyses. Thus, future trials comparing alternative IPT regimens may benefit from mediation analyses. Additionally, our approach informs future studies that will investigate the pathways through which IPTp in infancy influences child growth and health.

Overall, the effects on child growth among multigravidae were similar in size or larger than effect sizes of preventive nutritional interventions. IPTp-SP increased mean LAZ by up to 0.27 Z compared to DP, and DP increased mean WLZ by up to 0.28 Z compared to SP. These differences in Z are similar to effect sizes for infant multiple micronutrient supplements in the exclusive breastfeeding stage.^40^ Many other early life nutritional interventions, such as nutrition education and counseling,^41^ complementary feeding,^41^ maternal micronutrient supplements,^40^ and small quantity lipid-based nutrient supplements, have had smaller effects.^42^ Importantly, we observed benefits of IPTp to child growth during the exclusive breastfeeding stage, when growth faltering onset is high and few nutritional interventions are delivered.^5^^,^^6^

Certain inflammation-related proteins mediated the effects of IPTp on growth, with the strongest evidence for IL-18 as a potential mediator of DP’s effect on improved WLZ. While direct links between IL-18 and child growth have not been extensively studied, our findings contribute to the growing body of evidence that maternal inflammation influences foetal and infant growth, positing IL-18 as a protein warranting further investigation. IL-18 is a proinflammatory cytokine that is up-regulated during *Pf* malaria infection^43^^,^^44^; it is present throughout pregnancy in the placenta^45^ and in the blood, where levels become elevated in labour.^46^ Increased levels of IL-18 are associated with early pregnancy loss, recurrent miscarriages and other pregnancy complications.^45^, ^46^, ^47^ The positive effects of DP on child growth may be related to the reduction of this proinflammatory protein linked to adverse at-birth complications, which may suggest that maternal IL-18’s harmful effects extend beyond birth. Some studies suggest that maternal inflammation may impact infant growth outcomes,^27^^,^^48^^,^^49^ and studies have also shown that certain early infant biomarkers correlate with infant growth trajectories (e.g., insulin-like growth factor, systemic inflammation-related factors),^50^^,^^51^ suggesting a need to further elucidate the mechanisms connecting maternal inflammation to infant hormone and inflammation levels.

Potential mediating pathways not investigated in this study could also explain the observed differences in child growth between SP and DP. A separate analysis of this trial suggested that some of SP’s benefit may result from reduced febrile respiratory infections.^52^ Other studies point to SP’s effects on maternal nutrition: a trial found that SP increased maternal mid-upper arm circumference relative to DP,^53^ and an in vitro study found that SP improved nutrient absorption.^54^ Another trial identified gestational weight change as a mediator of the effect of IPTp-SP compared to DP.^24^ We did not observe mediation by gestational weight change, possibly because weight changes were relatively small in our study. In future studies, it would also be valuable to investigate whether SP’s antibiotic properties influence the vaginal or gut microbiome and whether SP modulates maternal immunity.^19^

Limitations include the relatively low incidence of stunting and wasting, particularly at older ages, which may have limited statistical power. Results among infants born to primigravid women may reflect a Type II error given that only 195 out of 782 enrolled women were primigravid. Second, we only had data on inflammation-related proteins in a subsample of mothers at delivery, which could have confounded our findings due to the extensive inflammation that occurs during labour and delivery. Inflammation earlier in pregnancy may also be important. Third, it is possible that mediator-outcome models were subject to residual unmeasured confounding; some possible confounders, such as maternal diet or nutritional status, were not measured in the original trial. Fourth, we investigated mediators individually, which implicitly assumed that mediators operated independently; it is possible that some mediators operated jointly. Fifth, results may not generalise to populations with differing sociodemographic factors, entomologic factors, co-intervention coverage, co-infections, parasite resistance, or transmission intensity. Sixth, infants lost to follow-up by age 12 months were more likely to be born to primigravidae and to have had placental malaria or low birth weight; thus, our study sample may underrepresent the most vulnerable infants in this population. Finally, this was an exploratory study that was not planned within the original trial protocol; however, a strength is that the statistical analysis was pre-specified, which can reduce confirmation bias.

This exploratory analysis indicates potential differences in how different IPTp regimens may affect infant growth in high malaria transmission settings from birth up to 8 months, a period with limited effective nutritional interventions. While current WHO IPTp policies primarily focus on low birth weight, our findings suggest that different IPTp regimens might influence ponderal and linear growth beyond birth. In areas of high parasite resistance to SP, our findings provide initial evidence that SP and DP could improve infant growth through distinct mechanisms. However, given the exploratory nature of this study, further research is needed to assess the potential impact of combined SP and DP on birth outcomes and child growth for enhanced public health impact compared to either regimen alone.

## Contributors

JBC and PJ designed the study and obtained funding for this analysis. GD, AK, and PJ contributed to data collection in the original trial. JBC, SH, KR, and PJ developed the statistical analysis plan. JBC, YT, SH, and KR analysed the data. YT, AN, KR, and JBC wrote the first draft of the manuscript. All authors reviewed and approved the final manuscript. YT and JBC accessed and verified the underlying data.

## Data sharing statement

Primary data were collected by clinicians on case record forms and double entered into a Microsoft Access Database by trained data personnel. Data from the original trial is available at https://clinepidb.org/ce/app/workspace/analyses/DS_8786631aaf/new/variables/EUPATH_0000096/EUPATH_0015457. Replication scripts are available at https://github.com/YanweiTong-Iris/IPTp-BC3-mediation/tree/main.

## Declaration of interests

Anna Nguyen is the recipient of a grant from them National Institute of Allergy and Infectious Diseases (F31AI179107) focused on evaluating the causal effects of anti-malarial chemoprevention in pregnancy and childhood on growth outcomes using a different dataset. The other authors declare no further potential conflicts of interest.
