# Supplementary material for: Intermittent preventive treatment for malaria in pregnancy and infant growth: a mediation analysis of a randomised trial
Source: eBioMedicine. 2024 Oct 16;109:105397. doi: 10.1016/j.ebiom.2024.105397 (PMC11530852; doi:10.1016/j.ebiom.2024.105397)
Supplement: Supplement S1 and S2, Supplementary Figs. S1–S13, and Tables S1–S5 [file mmc1.docx]

**SUPPLEMENTARY**

**
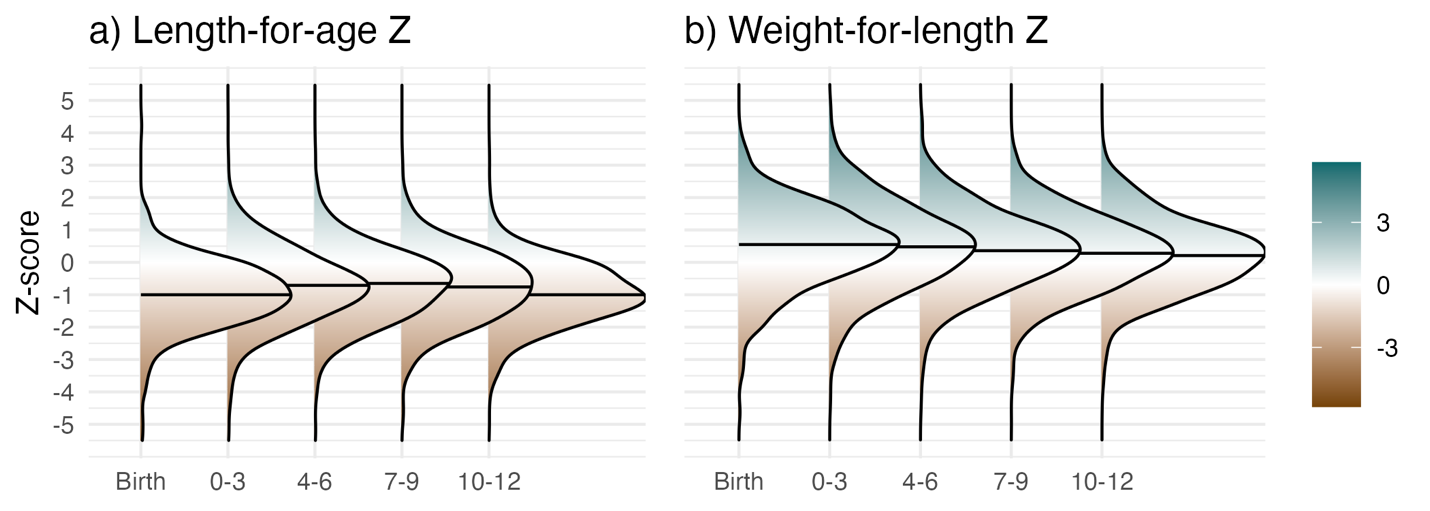
**

**Figure S1. Child growth from birth through 12 months**

0-3 includes children aged 1 day to 3 months, 4-6 includes children aged >3 to 6 months, 7-9 includes children aged >6 to 9 months, and 10-12 includes children aged >9 to 12 months. In a) and b) horizontal lines in each density plot indicate the median Z-score for each age category. Panel a) and b) include data from N=633 children at birth, N=624 from 1 day-3 months, N=590 from >3-6 months, N=570 from >6-9 months, and N=558 from >9-12 months. Panel c) includes data from N=633 children at birth, N=522 from 1 day-3 months, N=379 from >3-6 months, N=335 from >6-9 months, and N=300 from >9-12 months. Panel d) includes data from N=633 children at birth, N=607 from 1 day-3 months, N=501 from >3-6 months, N=469 from >6-9 months, and N=447 from >9-12 months. ****

**Figure S2 Total effect of IPTp DP vs. SP on mean child growth Z-scores corrected for gestational age by child age and gravidity**

Includes 630 children measured from birth to 12 months. Excludes children with negative ages after gestational age correction.

**
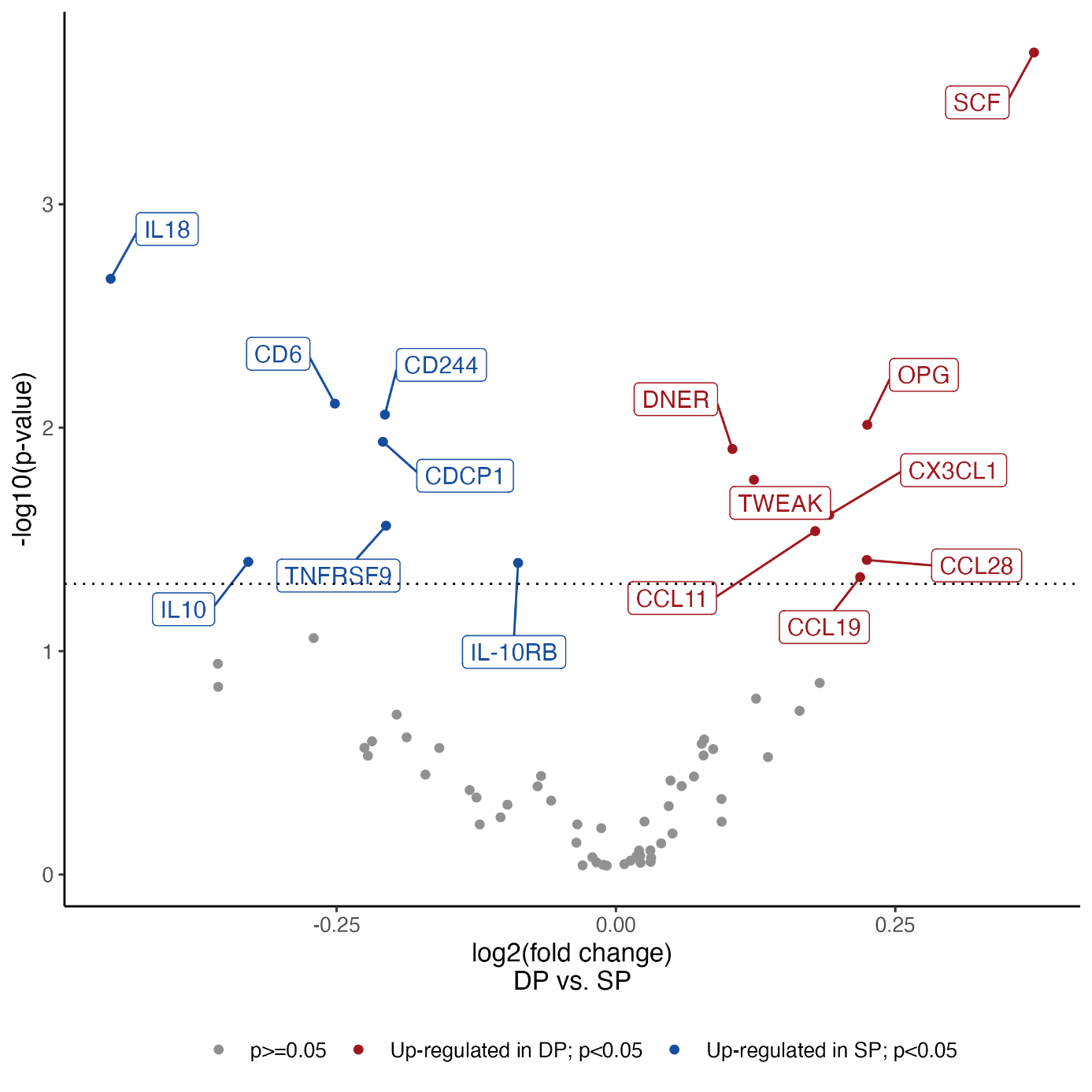
**

**Figure S3. Volcano plot of maternal inflammation-related proteins at delivery by IPTp-DP vs. IPTp-SP among all gravidae**

Red points represent proteins upregulated in DP with p<0.05, blue points represent proteins upregulated in SP with p<0.05, and grey points represent non-differentially expressed proteins. Colour coding in the plot is based on p-values that were not corrected for multiple testing. After the Benjamini-Hochberg correction (false discovery rate p-value < 0.05), only SCF had a p-value < 0.05. Among all gravidae, proteins upregulated in SP were IL18, CD6, CD244, CDCP1, TNFRSF9, IL10, and IL-10RB; proteins upregulated in DP were SCF, OPG, DNER, TWEAK, CX3CL1, CCL11, CCL28, and CCL19. Note that among primigravidae (sample size=51), proteins CD5 and ADA were upregulated in SP, which were not shown on this volcano plot. Includes N$\in$ [249, 250] children with data from Olink analyses.

Inflammation protein names can be found in Table S1.

**
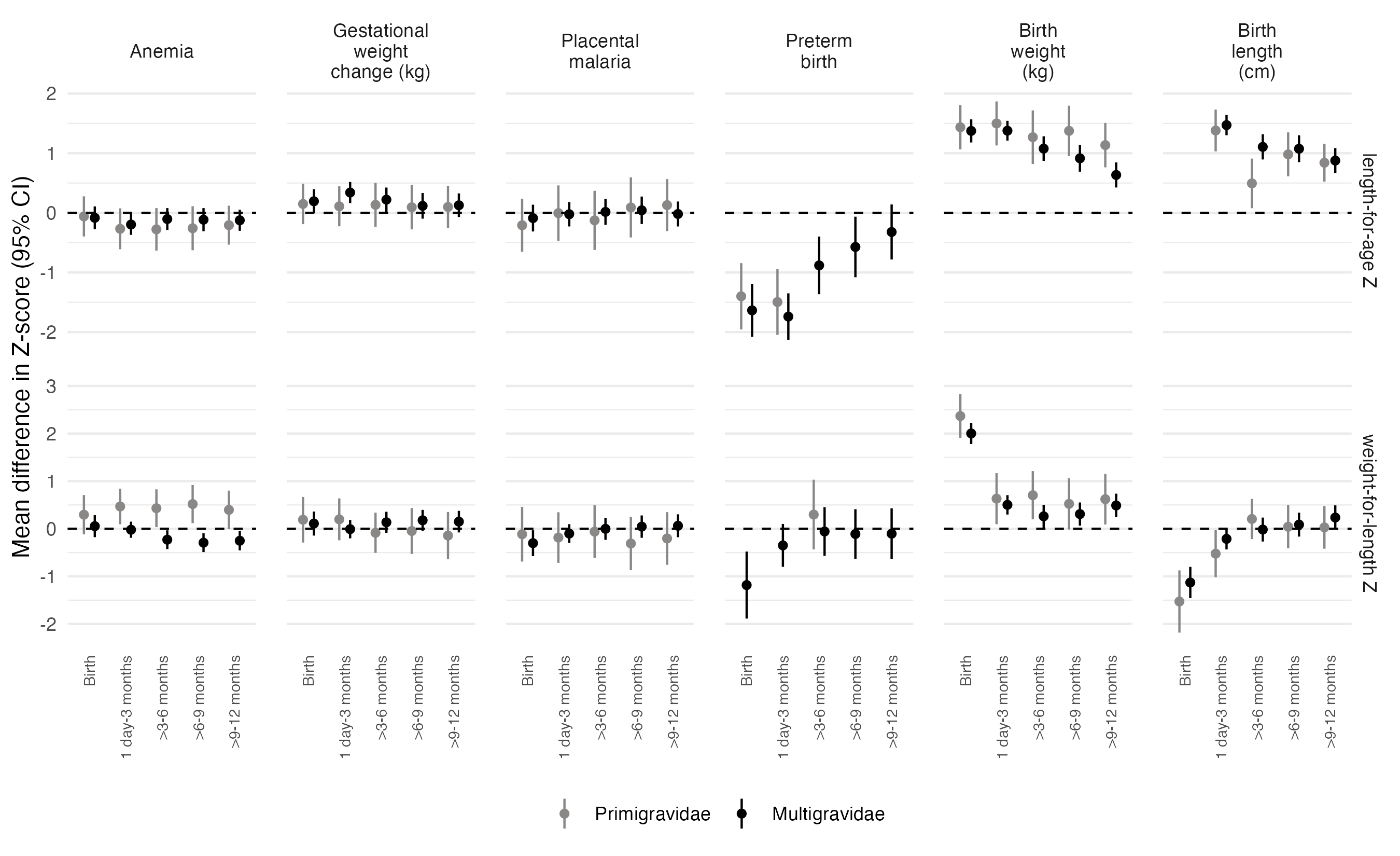
**

**Figure S4. Associations between potential non-inflammation-related mediators and mean child growth Z-scores**

Mean differences in Z-scores between each mediator and mean Z-scores adjusted by infant sex, maternal age, maternal baseline parasitaemia, gestational age at enrolment, gravidity, maternal education, and household wealth.

Note: Gestational weight change and birth length values have been rescaled (multiplied by a factor of 5) to enhance clarity and visualisation on the plot. This adjustment aligns their y-axis range with the other mediators for a more visually coherent presentation. Includes data from N$\in$ [555, 633] children at birth, N$\in$ [545, 620] from 1 day-3 months, N$\in$ [538, 587] from >3-6 months, N$\in$ [525, 570] from >6-9 months, and N$\in$ [515, 558] from >9-12 months.

**
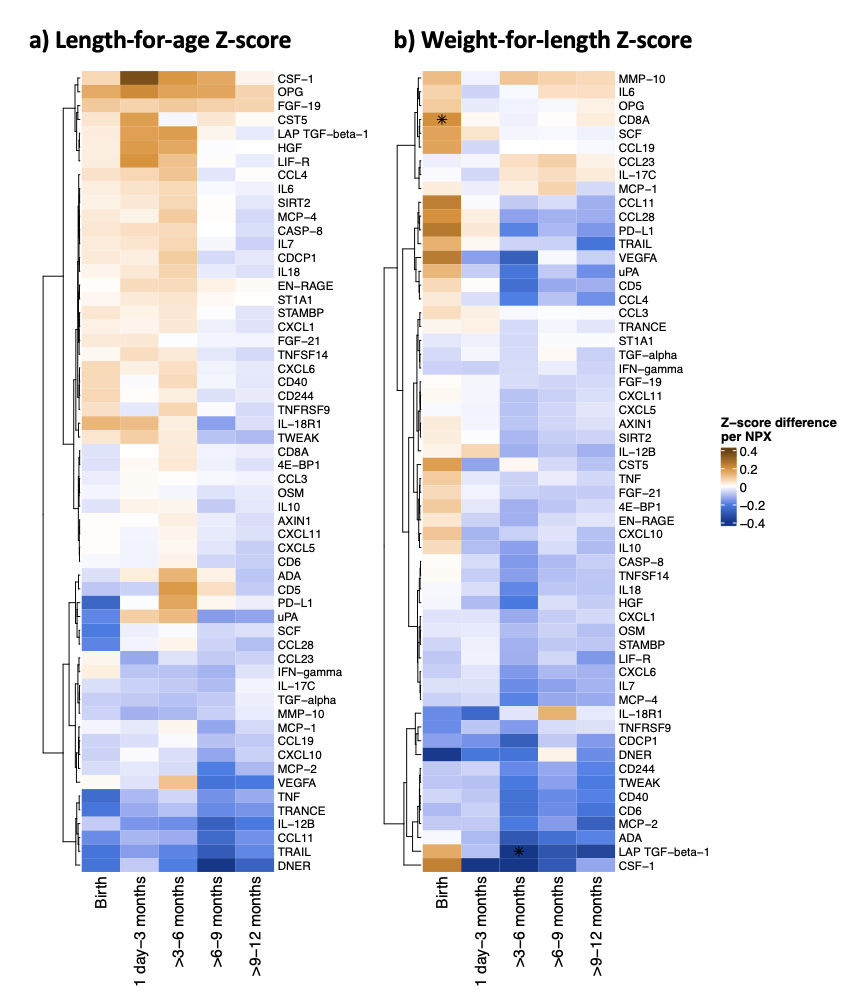
**

**Figure S5 Associations between maternal inflammation-related proteins at delivery and mean child growth Z-scores**

*

*

Mean Z-score differences per inflammation-related protein NPX were estimated with a model adjusted for infant sex, maternal age, gravidity, maternal baseline parasitaemia, gestational age at enrolment, maternal education, household wealth. Panel a) includes data from N=255 children at birth, N=251 from 1 day-3 months, N=255 from >3-6 months, N=255 from >6-9 months, and N=255>9-12 months. Panel b) includes data from N=245 children at birth, N=247 from 1 day-3 months, N=255 from >3-6 months, N=254 from >6-9 months, and N=255 from >9-12 months.

*Statistically significant among all gravidae after Benjamini-Hochberg correction (false discovery rate p-value < 0.05).

Inflammation protein names can be found in Table S1.

**
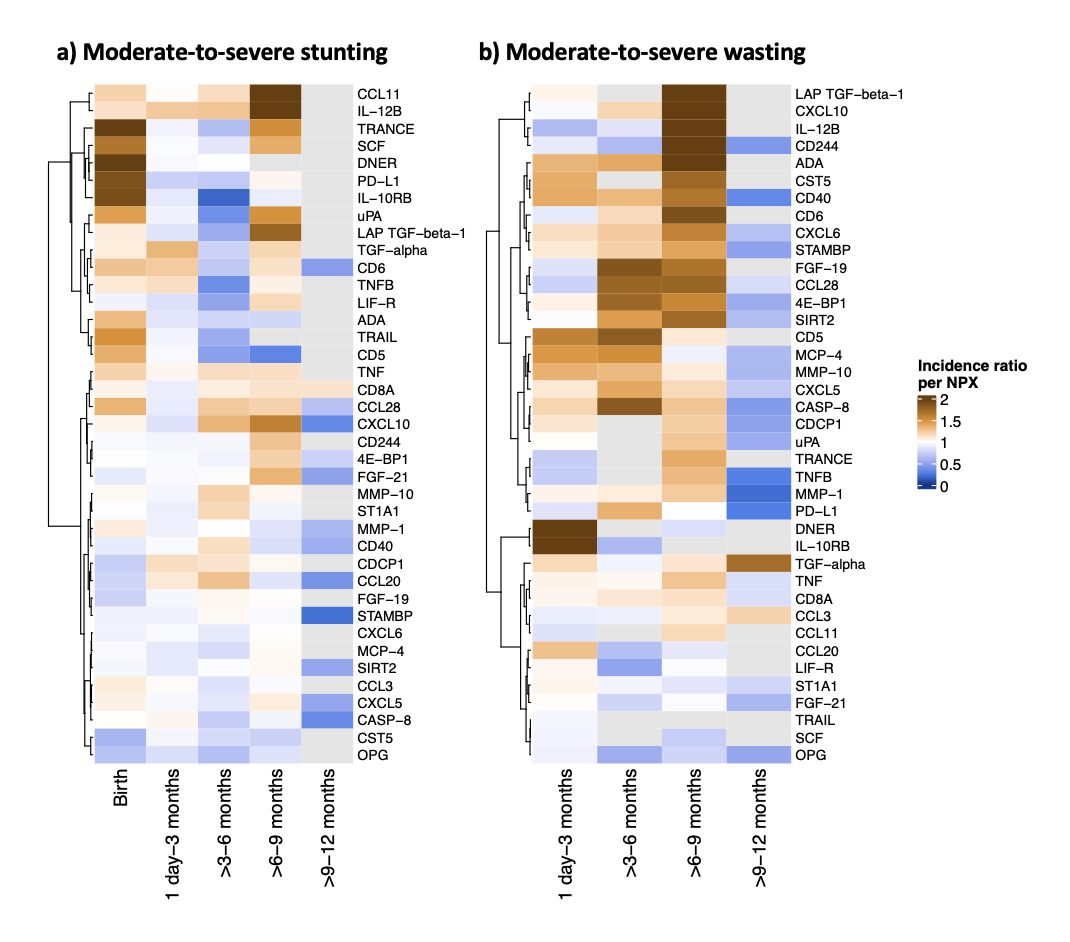
**

**Figure S6 Associations between potential inflammation-related protein mediators and stunting and wasting**

Incidence ratios per inflammation-related protein NPX were estimated with a model adjusted by infant sex, primigravida, maternal age, maternal baseline parasitaemia, gestational age at enrolment, maternal education, and household wealth. The reference group was SP. Missing point estimates were due to data sparsity and were filled as grey. When there were fewer than 10 incident cases in a certain age group, models were not fit. Panel a) includes data from N $\in$ [254, 255] children at birth, N $\in$ [210, 211] from 1 day-3 months, N $\in$ [159, 160] from >3-6 months, N=148 from >6-9 months, and N=136>9-12 months. Panel b) includes data from N $\in$ [245, 246] from 1 day-3 months, N $\in$ [223, 224] from >3-6 months, N $\in$ [217, 218] from >6-9 months, and N $\in$ [209, 210] from >9-12 months.

Inflammation protein names can be found in Table S1.

| **Mediator (M)** | **I → M** |  | **M → LAZ** | **I → M → LAZ** |  | **M → WLZ** | **I → M → WLZ** |
| --- | --- | --- | --- | --- | --- | --- | --- |
| Anaemia |  |  |  |  |  |  |  |
| Gestational weight change |  |  | ↑ |  |  |  |  |
| Placental malaria | DP |  |  |  |  | ↓ | DP |
| Pre-term birth |  |  | ↓ |  |  | ↓ |  |
| Birth length | SP |  | ↑ | SP |  | ↑ | DP |
| Birth weight | SP |  | ↑ | SP |  | ↑ | SP |
| CDCP1 | SP |  |  |  |  | ↓ | DP |
| CD6 | SP |  |  |  |  | ↓ | DP |
| IL18 | SP |  |  |  |  | ↓ | DP |
| SCF | DP |  | ↓ | SP |  |  |  |
| DNER | DP |  | ↓ | SP |  |  |  |
| OPG | DP |  | ↑ | DP |  |  |  |

| DP | DP promotes health / protein is up in DP |
| --- | --- |
| SP | SP promotes health / protein is up in SP |

**Figure S7. Summary of results for each mediating pathway**

I: Intervention (IPTp DP vs. SP); LAZ: length-for-age Z; WLZ: weight-for-length Z

I → M indicate results of intervention-mediator models. M → LAZ indicate results of models of the association between each mediator and LAZ (and analogously for WLZ). I → M→ LAZ indicate results of mediation models for LAZ (and analogously for WLZ). Cells with DP indicate that IPTp-DP improved the outcome compared to IPT-SP or that DP increased inflammation-related proteins relative to SP. We considered improvements to be reductions in anaemia, placental malaria, pre-term birth, and inflammation-related proteins and increases in gestational weight change and birth length or weight. Arrows indicate whether the mediator was associated with higher (↑) or lower (↓) mean Z-scores.

Inflammation protein names can be found in Table S1.

**
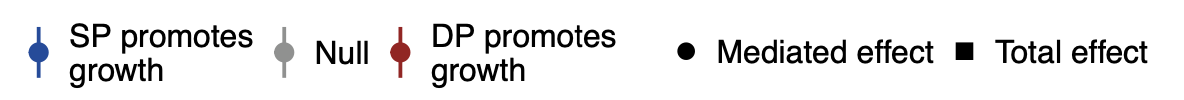
**

**Figure S8. Total effects and mediated effects on incidences of child stunting and wasting**

The total effects compare incidence of stunting and wasting between IPTp-DP and IPTp-SP using unadjusted models. The mediated effects were adjusted by infant sex, maternal age, maternal baseline parasitaemia, gestational age at enrolment, gravidity, maternal education, and household wealth. The reference group was SP. Note: Missing point estimates were due to data sparsity. When there were fewer than 5 incident cases or 5 observed values of a binary mediator in a certain age group, models were not fit.

For non-Olink mediators, the mediated effects in panel a) includes data from N = 622 children at birth, N $\in$ [505, 507] from 1 day-3 months, N $\in$ [378, 379] from >3-6 months, and N $\in$ [333, 334] from >6-9 months; the mediated effects in panel b) includes data from N=596 children at birth, N $\in$ [575, 577] from 1 day-3 months, N $\in$ [496, 497] from >3-6 months, N $\in$ [467, 468] from >6-9 months, and N $\in$ [447, 448] from >9-12 months. For the Olink mediator SCF, the mediated effects in panel a) includes data from N=255 children at birth, N=211 from 1 day-3 months, and N=160 from >3-6 months. The sample sizes for total effects can be found in Table 2. Inflammation protein names can be found in Table S1.

**
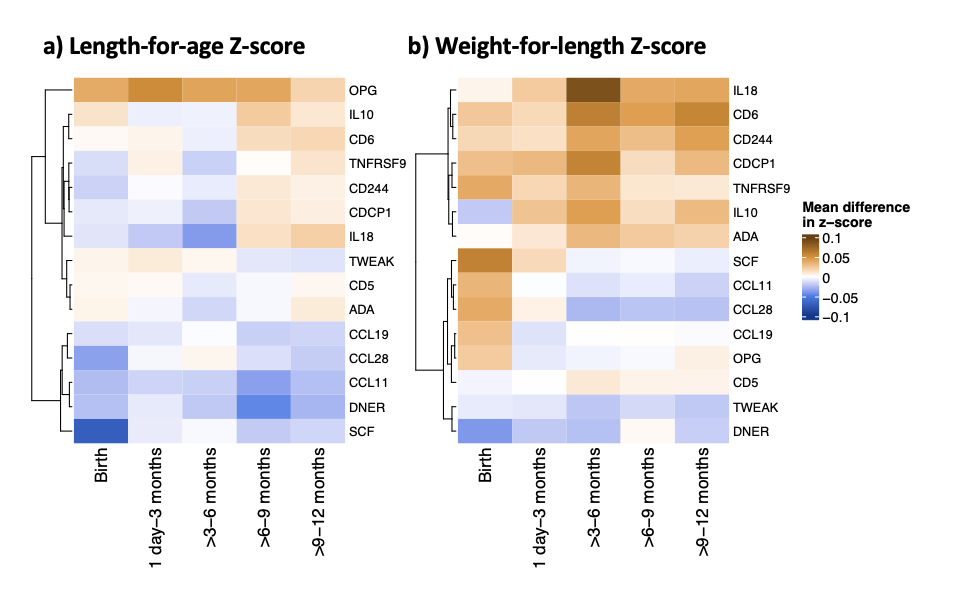
**

**Figure S9 Inflammation-related protein-mediated effects of IPTp-DP vs. IPTp-SP on Z-scores**

Mediated effects were adjusted by infant sex, primigravida, maternal age, maternal baseline parasitaemia, gestational age at enrolment, maternal education, and household wealth. Analysis includes all gravidae. The reference group was SP. Panel a) includes data from N=255 children at birth, N=251 from 1 day-3 months, N=255 from >3-6 months, N=255 from >6-9 months, and N=255>9-12 months. Panel b) includes data from N=245 children at birth, N=247 from 1 day-3 months, N=255 from >3-6 months, N=254 from >6-9 months, and N=255>9-12 months. Inflammation protein names can be found in Table S1.

**Figure S10 Inflammation-related protein-mediated effects on length-for-age Z**

Mediated effects were adjusted for infant sex, maternal age, gravidity, maternal baseline parasitaemia, gestational age at enrolment, maternal education, and household wealth. Analysis includes all gravidae. The reference group was SP. Includes data from N=255 children at birth, N=251 from 1 day-3 months, N=255 from >3-6 months, N=255 from >6-9 months, and N=255 from>9-12 months.

Inflammation protein names can be found in Table S1.

**Figure S11 Inflammation-related protein-mediated effects on child stunting**

Mediated effects were adjusted by infant sex, gravidity, maternal age, maternal baseline parasitaemia, gestational age at enrolment, maternal education, and household wealth. Mediation models were only fit from birth through age 6 months due to data sparsity at other ages. Analysis includes all gravidae. The reference group was SP. Includes data from N=255 children at birth, N=211 from 1 day-3 months, and N=160 from >3-6 months. Inflammation protein names can be found in Table S1.

**Figure S12 Inflammation-related protein-mediated effects on weight-for-length Z**

Mediated effects were adjusted by infant sex, gravidity, maternal age, maternal baseline parasitaemia, gestational age at enrolment, maternal education, and household wealth. Analysis includes all gravidae. The reference group was SP. Includes data from N=245 children at birth, N=247 from 1 day-3 months, N=255 from >3-6 months, N=254 from >6-9 months, and N=255 from >9-12 months.

Inflammation protein names can be found in Table S1.

**Figure S13 Inflammation-related protein-mediated effects on child wasting**

Mediated effects were adjusted by infant sex, gravidity, maternal age, maternal baseline parasitaemia, gestational age at enrolment, maternal education, and household wealth. Analysis includes all gravidae. The reference group was SP. Mediation models did not fit at birth or >9-12 months due to data sparsity. Includes data from N=246 children from 1 day-3 months, N=224 from >3-6 months, and N=218 from >6-9 months. Inflammation protein names can be found in Table S1.

**Table S1.** Olink Target 96 Inflammation - Protein Reference Chart

| **UniProt ID** | **Gene** | **Protein name** |
| --- | --- | --- |
| **Q13541** | 4E-BP1 (EIF4EBP1) | Eukaryotic translation initiation factor 4E-binding protein 1 |
| **P00813** | ADA | Adenosine deaminase |
| **Q5T4W7** | ARTN | Artemin |
| **O15169** | AXIN1 | Axin-1 |
| **P01138** | Beta-NGF (NGF) | Beta-nerve growth factor |
| **Q14790** | CASP8 | Caspase-8 |
| **P10147** | CCL3 | C-C motif chemokine 3 |
| **P13236** | CCL4 | C-C motif chemokine 4 |
| **P51671** | CCL11 | Eotaxin |
| **Q99731** | CCL19 | C-C motif chemokine 19 |
| **P78556** | CCL20 | C-C motif chemokine 20 |
| **P55773** | CCL23 | C-C motif chemokine 23 |
| **O15444** | CCL25 | C-C motif chemokine 25 |
| **Q9NRJ3** | CCL28 | C-C motif chemokine 28 |
| **P06127** | CD5 | T-cell surface glycoprotein CD5 |
| **P30203** | CD6 | T cell surface glycoprotein CD6 isoform |
| **P01732** | CD8A | T-cell surface glycoprotein CD8 alpha chain |
| **P25942** | CD40 | CD40L receptor |
| **Q9BZW8** | CD244 | Natural killer cell receptor 2B4 |
| **Q9H5V8** | CDCP1 | CUB domain-containing protein 1 |
| **P09603** | CSF-1 | Macrophage colony-stimulating factor 1 |
| **P28325** | CST5 | Cystatin D |
| **P78423** | CX3CL1 | Fractalkine |
| **P09341** | CXCL1 | C-X-C motif chemokine 1 |
| **P42830** | CXCL5 | C-X-C motif chemokine 5 |
| **P80162** | CXCL6 | C-X-C motif chemokine 6 |
| **Q07325** | CXCL9 | C-X-C motif chemokine 9 |
| **P02778** | CXCL10 | C-X-C motif chemokine 10 |
| **O14625** | CXCL11 | C-X-C motif chemokine 11 |
| **Q8NFT8** | DNER | Delta and Notch-like epidermal growth factor-related receptor |
| **P80511** | EN-RAGE (S100A12) | Protein S100-A12 |
| **P12034** | FGF-5 | Fibroblast growth factor 5 |
| **O95750** | FGF-19 | Fibroblast growth factor 19 |
| **Q9NSA1** | FGF-21 | Fibroblast growth factor 21 |
| **Q9GZV9** | FGF-23 | Fibroblast growth factor 23 |
| **P49771** | Flt3L (FLT3LG) | Fms-related tyrosine kinase 3 ligand |
| **P39905** | GDNF | Glial cell line-derived neurotrophic factor |
| **P14210** | HGF | Hepatocyte growth factor |
| **P01579** | IFN-gamma (IFNG) | Interferon gamma |
| **P01583** | IL-1 alpha | Interleukin-1 alpha |
| **P60568** | IL-2 | Interleukin-2 |
| **P14784** | IL-2RB | Interleukin-2 receptor subunit beta |
| **P05112** | IL-4 | Interleukin-4 |
| **P05113** | IL5 | Interleukin-5 |
| **P05231** | IL6 | Interleukin-6 |
| **P13232** | IL-7 | Interleukin-7 |
| **P10145** | IL-8 (CXCL8) | Interleukin-8 |
| **P22301** | IL10 | Interleukin-10 |
| **Q13651** | IL-10RA | Interleukin-10 receptor subunit alpha |
| **Q08334** | IL-10RB | Interleukin-10 receptor subunit beta |
| **P29460** | IL-12B | Interleukin-12 subunit beta |
| **P35225** | IL-13 | Interleukin-13 |
| **Q13261** | IL-15RA | Interleukin-15 receptor subunit alpha |
| **Q16552** | IL-17A | Interleukin-17A |
| **Q9P0M4** | IL-17C | Interleukin-17C |
| **Q14116** | IL-18 | Interleukin-18 |
| **Q13478** | IL-18R1 | Interleukin-18 receptor 1 |
| **Q9NYY1** | IL-20 | Interleukin-20 |
| **Q9UHF4** | IL-20RA | Interleukin-20 receptor subunit alpha |
| **Q8N6P7** | IL-22 RA1 | Interleukin-22 receptor subunit alpha-1 |
| **Q13007** | IL-24 | Interleukin-24 |
| **O95760** | IL-33 | Interleukin-33 |
| **P15018** | LIF | Leukemia inhibitory factor |
| **P42702** | LIF-R | Leukemia inhibitory factor receptor |
| **P13500** | MCP-1 (CCL2) | Monocyte chemotactic protein 1 |
| **P80075** | MCP-2 (CCL8) | Monocyte chemotactic protein 2 |
| **P80098** | MCP-3 (CCL7) | Monocyte chemotactic protein 3 |
| **Q99616** | MCP-4 (CCL13) | Monocyte chemotactic protein 4 |
| **P03956** | MMP-1 | Matrix metalloproteinase-1 |
| **P09238** | MMP-10 | Matrix metalloproteinase-10 |
| **Q99748** | NRTN | Neurturin |
| **P20783** | NT-3 (NTF3) | Neurotrophin-3 |
| **O00300** | OPG | Osteoprotegerin |
| **P13725** | OSM | Oncostatin-M |
| **Q9NZQ7** | PD-L1 (CD274) | Programmed cell death 1 ligand 1 |
| **P21583** | SCF | Stem cell factor |
| **Q8IXJ6** | SIRT2 | SIR2-like protein 2 |
| **Q13291** | SLAMF1 | Signalling lymphocytic activation molecule |
| **P50225** | ST1A1 (SULT1A1) | Sulfotransferase 1A1 |
| **O95630** | STAMBP | STAM-binding protein |
| **P01135** | TGF-alpha (TGFA) | Transforming growth factor alpha |
| **P01137** | LAP TGF-beta-1 (TGFB1) | Latency-associated peptide transforming growth factor beta-1 |
| **P01375** | TNF | Tumour necrosis factor |
| **P01374** | TNFB (LTA) | TNF-beta |
| **Q07011** | TNFRSF9 | Tumour necrosis factor receptor superfamily member 9 |
| **O43557** | TNFSF14 | Tumour necrosis factor ligand superfamily member 14 |
| **P50591** | TRAIL (TNFSF10) | TNF-related apoptosis-inducing ligand |
| **O14788** | TRANCE (TNFSF11) | TNF-related activation-induced cytokine |
| **Q969D9** | TSLP | Thymic stromal lymphopoietin |
| **43508** | TWEAK (TNFSF12) | Tumour necrosis factor ligand superfamily member 12 |
| **P00749** | uPA (PLAU) | Urokinase-type plasminogen activator |
| **P15692** | VEGFA | Vascular endothelial growth factor A |

**Table S2. Inflammation-related proteins included in each analysis**

|  | **Passed LOD* check** | **Significant† intervention-mediator results** | **Significant† mediator-outcome results** | **Included in the mediation analysis** |
| --- | --- | --- | --- | --- |
| **IL8** | √ |  |  |  |
| **VEGFA** | √ |  | √ |  |
| **CD8A** | √ |  | √ |  |
| **MCP-3** |  |  |  |  |
| **GDNF** |  |  |  |  |
| **CDCP1** | √ | √ | √ | √ |
| **CD244** | √ | √ | √ | √ |
| **IL7** | √ |  | √ |  |
| **OPG** | √ | √ | √ | √ |
| **LAP TGF-beta-1** | √ |  | √ |  |
| **uPA** | √ |  | √ |  |
| **IL6** | √ |  | √ |  |
| **IL-17C** | √ |  | √ |  |
| **MCP-1** | √ |  | √ |  |
| **IL-17A** |  |  |  |  |
| **CXCL11** | √ |  | √ |  |
| **AXIN1** | √ |  | √ |  |
| **TRAIL** | √ |  | √ |  |
| **IL-20RA** |  |  |  |  |
| **CXCL9** | √ |  |  |  |
| **CST5** | √ |  | √ |  |
| **IL-2RB** |  |  |  |  |
| **IL-1 alpha** |  |  |  |  |
| **OSM** | √ |  | √ |  |
| **IL2** |  |  |  |  |
| **CXCL1** | √ |  | √ |  |
| **TSLP** |  |  |  |  |
| **CCL4** | √ |  | √ |  |
| **CD6** | √ | √ | √ | √ |
| **SCF** | √ | √ | √ | √ |
| **IL18** | √ | √ | √ | √ |
| **SLAMF1** |  |  |  |  |
| **TGF-alpha** | √ |  | √ |  |
| **MCP-4** | √ |  | √ |  |
| **CCL11** | √ | √ | √ | √ |
| **TNFSF14** | √ |  | √ |  |
| **FGF-23** |  |  |  |  |
| **IL-10RA** |  |  |  |  |
| **FGF-5** |  |  |  |  |
| **MMP-1** | √ |  |  |  |
| **LIF-R** | √ |  | √ |  |
| **FGF-21** | √ |  | √ |  |
| **CCL19** | √ | √ | √ | √ |
| **IL-15RA** |  |  |  |  |
| **IL-10RB** | √ | √ |  |  |
| **IL-22 RA1** |  |  |  |  |
| **IL-18R1** | √ |  | √ |  |
| **PD-L1** | √ |  | √ |  |
| **Beta-NGF** |  |  |  |  |
| **CXCL5** | √ |  | √ |  |
| **TRANCE** | √ |  | √ |  |
| **HGF** | √ |  | √ |  |
| **IL-12B** | √ |  | √ |  |
| **IL-24** |  |  |  |  |
| **IL13** |  |  |  |  |
| **ARTN** |  |  |  |  |
| **MMP-10** | √ |  | √ |  |
| **IL10** | √ | √ | √ | √ |
| **TNF** | √ |  | √ |  |
| **CCL23** | √ |  | √ |  |
| **CD5** | √ | √ | √ | √ |
| **CCL3** | √ |  | √ |  |
| **Flt3L** | √ |  |  |  |
| **CXCL6** | √ |  | √ |  |
| **CXCL10** | √ |  | √ |  |
| **4E-BP1** | √ |  | √ |  |
| **IL-20** |  |  |  |  |
| **SIRT2** | √ |  | √ |  |
| **CCL28** | √ | √ | √ | √ |
| **DNER** | √ | √ | √ | √ |
| **EN-RAGE** | √ |  | √ |  |
| **CD40** | √ |  | √ |  |
| **IL33** |  |  |  |  |
| **IFN-gamma** | √ |  | √ |  |
| **FGF-19** | √ |  | √ |  |
| **IL4** |  |  |  |  |
| **LIF** |  |  |  |  |
| **NRTN** |  |  |  |  |
| **MCP-2** | √ |  | √ |  |
| **CASP-8** | √ |  | √ |  |
| **CCL25** | √ |  |  |  |
| **CX3CL1** | √ | √ |  |  |
| **TNFRSF9** | √ | √ | √ | √ |
| **NT-3** |  |  |  |  |
| **TWEAK** | √ | √ | √ | √ |
| **CCL20** | √ |  |  |  |
| **ST1A1** | √ |  | √ |  |
| **STAMBP** | √ |  | √ |  |
| **IL5** |  |  |  |  |
| **ADA** | √ | √ | √ | √ |
| **TNFB** | √ |  |  |  |
| **CSF-1** | √ |  | √ |  |

* LOD: limit of detection

**†** p-value not accounting for multiple testing < 0.05

Inflammation protein names can be found in Table S1

**Table S3. Baseline characteristics of participants with and without complete infant follow-up through age 12 months**

|  | **Complete follow-up (n=537)** | **Incomplete follow-up (n=96)** |
| --- | --- | --- |
| **Maternal characteristics at baseline** |  |  |
| Mother’s age at enrolment (years) | 24 (19 – 28) | 21 (18 – 26) |
| Gestational age at enrolment (weeks) | 15.4 (13.4 – 17.6) | 15.7 (13.9 – 17.6) |
| Primigravidae | 117 (22%) | 35 (36%) |
| Parasite prevalence by microscopy or qPCR at enrolment | 439 (82%) | 82 (85%) |
| Haemoglobin concentration at enrolment (g/dL) | 11.5 (10.7 – 12.3) | 11.1 (10.4 – 12.2) |
| **Infant characteristics at birth** |  |  |
| Gestational age at delivery (weeks) | 39.9 (38.9 – 40.7) | 39.4 (38.6 – 40.6) |
| Preterm birth | 23 (4%) | 11 (11%) |
| Placental malaria* | 239 (45%) | 56 (59%) |
| Infant sex - Female | 277 (52%) | 46 (48%) |
| Low birth weight | 27 (5%) | 14 (15%) |

Data are median (IQR) or n (%).

* Placental malaria was determined by microscopy, qPCR, histopathological assessment, or loop-mediated isothermal amplification.

**Table S4. Associations between non-inflammation-related potential mediators and stunting and wasting**

|  | Stunting | | Wasting | |
| --- | --- | --- | --- | --- |
|  | N | Incidence ratio (95% CI) | N | Incidence ratio (95% CI) |
| Maternal anaemia |  |  |  |  |
| Birth | 608 | 1.07 (0.72, 1.59) | 582 | 0.43 (0.15, 1.24) |
| 1 day-3 months | 495 | 1.35 (0.93, 1.97) | 564 | 0.87 (0.54, 1.41) |
| >3-6 months | 374 | 0.83 (0.41, 1.65) | 485 | 1.48 (0.59, 3.69) |
| >6-9 months | 330 | 0.98 (0.45, 2.13) | 458 | 1.51 (0.47, 4.92) |
| >9-12 months | 296 | 0.14 (0.02, 1.11) | 437 | 2.58 (0.74, 9.03) |
|  |  |  |  |  |
| Gestational weight change (kg) |  |  |  |  |
| Birth | 567 | 0.94 (0.85, 1.03) | 555 | 1.05 (0.84, 1.32) |
| 1 day-3 months | 473 | 0.97 (0.89, 1.05) | 530 | 0.94 (0.84, 1.05) |
| >3-6 months | 361 | 0.94 (0.81, 1.09) | 463 | 0.99 (0.80, 1.23) |
| >6-9 months | 320 | 1.05 (0.88, 1.26) | 439 | 0.92 (0.71, 1.19) |
| >9-12 months | 287 | 0.93 (0.71, 1.21) | 419 | 1.01 (0.78, 1.33) |
|  |  |  |  |  |
| Placental malaria |  |  |  |  |
| Birth | 622 | 1.39 (0.86, 2.26) | 596 | 1.22 (0.37, 3.97) |
| 1 day-3 months | 507 | 0.92 (0.59, 1.46) | 577 | 1.00 (0.55, 1.81) |
| >3-6 months | 379 | 1.30 (0.59, 2.86) | 497 | 1.12 (0.35, 3.55) |
| >6-9 months | 334 | 1.21 (0.51, 2.88) | 468 | 0.82 (0.20, 3.38) |
| >9-12 months | 300 | 0.22 (0.02, 1.99) | 447 | 1.01 (0.23, 4.39) |
|  |  |  |  |  |
| Pre-term birth |  |  |  |  |
| Birth | 622 | 4.01 (2.38, 6.73) | 596 | 1.81 (0.23, 14.24) |
| 1 day-3 months | 507 | 2.13 (0.98, 4.62) | 577 | 2.58 (1.16, 5.72) |
| >3-6 months |  | -- |  | -- |
| >6-9 months |  | -- | 468 | 2.85 (0.35, 23.38) |
| >9-12 months |  | -- |  | -- |
|  |  |  |  |  |
| Birth length (cm) |  |  |  |  |
| Birth | 622 | 0.58 (0.53, 0.63 | 596 | 1.66 (1.34, 2.06) |
| 1 day-3 months | 505 | 0.8 (0.74, 0.87) | 575 | 1.08 (0.94, 1.24) |
| >3-6 months | 378 | 0.79 (0.67, 0.92) | 496 | 0.91 (0.74, 1.12) |
| >6-9 months | 334 | 0.79 (0.66, 0.94) | 467 | 1.21 (0.87, 1.68) |
| >9-12 months | 300 | 1.22 (0.87, 1.71) | 446 | 0.9 (0.72, 1.13) |
|  |  |  |  |  |
| Birth weight (kg) |  |  |  |  |
| Birth | 622 | 0.13 (0.08, 0.2) | 596 | 0.02 (0, 0.09) |
| 1 day-3 months | 522 | 0.28 (0.17, 0.47) | 577 | 0.3 (0.15, 0.6) |
| >3-6 months | 379 | 0.38 (0.15, 0.97) | 497 | 0.47 (0.13, 1.77) |
| >6-9 months | 335 | 0.27 (0.09, 0.78) | 468 | 3.30 (0.65, 16.68) |
| >9-12 months | 300 | 1.54 (0.26, 9.11) | 447 | 0.24 (0.04, 1.38) |

Incidence ratios for each potential mediator were adjusted by infant sex, primigravida, maternal age, maternal baseline parasitaemia, gestational age at enrolment, maternal education, and household wealth.

Note: missing values were due to data sparsity.

**Table S5. Mediated effects of IPTp-DP vs. IPTp-SP on Z-scores**

|  | Length-for-age Z | | Weight-for-length Z | |
| --- | --- | --- | --- | --- |
|  | N | Mean difference in Z (95% CI) | N | Mean difference in Z (95% CI) |
| Maternal anaemia |  |  |  |  |
| Birth | 608 | 0.0037 (-0.0077, 0.0225) | 582 | -0.0028 (-0.0229, 0.0103) |
| 1 day-3 months | 593 | 0.0084 (-0.0116, 0.0357) | 581 | -0.0025 (-0.0187, 0.0082) |
| >3-6 months | 575 | 0.0043 (-0.0099, 0.0244) | 574 | 0.0030 (-0.0091, 0.0219) |
| >6-9 months | 559 | 0.0049 (-0.0099, 0.0256) | 558 | 0.0045 (-0.0095, 0.0264) |
| >9-12 months | 548 | 0.0049 (-0.0109, 0.0245) | 548 | 0.0039 (-0.0106, 0.0239) |
|  |  |  |  |  |
| Gestational weight  change (kg) |  |  |  |  |
| Birth | 567 | -0.0007 (-0.0182, 0.0144) | 555 | -0.0007 (-0.0183, 0.013) |
| 1 day-3 months | 552 | -0.0036 (-0.0305, 0.0206) | 545 | -0.0002 (-0.0101, 0.0085) |
| >3-6 months | 541 | -0.002 (-0.0264, 0.0186) | 538 | -0.0006 (-0.0139, 0.0092) |
| >6-9 months | 526 | -0.0005 (-0.018, 0.0138) | 525 | -0.0006 (-0.0177, 0.0137) |
| >9-12 months | 515 | 0.0004 (-0.0161, 0.0157) | 515 | 0.0002 (-0.0132, 0.0112) |
|  |  |  |  |  |
| Placental malaria |  |  |  |  |
| Birth | 622 | 0.0358 (-0.0318, 0.1055) | 596 | 0.0903 (-0.002, 0.183) |
| 1 day-3 months | 607 | 0.0049 (-0.0625, 0.0691) | 594 | 0.0414 (-0.0285, 0.1114) |
| >3-6 months | 587 | 0.0003 (-0.075, 0.0708) | 586 | 0.0057 (-0.0706, 0.0805) |
| >6-9 months | 569 | -0.0152 (-0.0897, 0.0545) | 568 | 0.0024 (-0.0727, 0.0782) |
| >9-12 months | 558 | -0.0059 (-0.0745, 0.0588) | 558 | -0.0040 (-0.0778, 0.0691) |
|  |  |  |  |  |
| Pre-term birth |  |  |  |  |
| Birth | 622 | 0.0098 (-0.0645, 0.0909) | 596 | 0.0087 (-0.0252, 0.0511) |
| 1 day-3 months | 607 | 0.0097 (-0.0695, 0.0916) | 594 | 0.0005 (-0.0143, 0.0161) |
| >3-6 months | 587 | 0.0134 (-0.024, 0.0656) | 586 | -0.0013 (-0.0253, 0.0206) |
| >6-9 months | 569 | 0.0112 (-0.0217, 0.0569) | 568 | -0.0017 (-0.0229, 0.0147) |
| >9-12 months | 558 | 0.0087 (-0.0159, 0.0433) | 558 | -0.0033 (-0.0245, 0.0109) |
|  |  |  |  |  |
| Birth length (cm) |  |  |  |  |
| Birth | 622 | -0.2522 (-0.4108, -0.0899) | 596 | 0.0883 (0.0223, 0.1575) |
| 1 day-3 months | 605 | -0.1235 (-0.2265, -0.0319) | 592 | 0.0231 (0.0026, 0.0551) |
| >3-6 months | 585 | -0.0815 (-0.1562, -0.0184) | 584 | -0.0035 (-0.026, 0.017) |
| >6-9 months | 567 | -0.0801 (-0.1586, -0.0099) | 566 | -0.0050 (-0.0254, 0.0123) |
| >9-12 months | 557 | -0.0620 (-0.1269, -0.0036) | 557 | -0.0125 (-0.0384, 0.0027) |
|  |  |  |  |  |
| Birth weight (kg) |  |  |  |  |
| Birth | 622 | -0.1115 (-0.2032, -0.023) | 596 | -0.149 (-0.2817, -0.0167) |
| 1 day-3 months | 607 | -0.1119 (-0.2058, -0.0207) | 594 | -0.038 (-0.0786, -0.0045) |
| >3-6 months | 587 | -0.0695 (-0.1444, 0.0009) | 586 | -0.0189 (-0.0499, 0.0037) |
| >6-9 months | 569 | -0.0623 (-0.1333, 0.0031) | 568 | -0.0222 (-0.0541, 0.0005) |
| >9-12 months | 558 | -0.0421 (-0.0943, 0.0059) | 558 | -0.0293 (-0.0707, 0.0036) |
|  |  |  |  |  |
| CD6 |  |  |  |  |
| Birth | 255 | 0.0026 (-0.0485, 0.0519) | 245 | 0.0247 (-0.0221, 0.0853) |
| 1 day-3 months | 251 | 0.0047 (-0.0447, 0.0561) | 247 | 0.0173 (-0.0257, 0.0734) |
| >3-6 months | 255 | -0.0056 (-0.0651, 0.049) | 255 | 0.0609 (0.0037, 0.1464) |
| >6-9 months | 255 | 0.0158 (-0.0361, 0.0765) | 254 | 0.0440 (-0.0056, 0.1172) |
| >9-12 months | 255 | 0.0183 (-0.0237, 0.0704) | 255 | 0.0575 (0.0032, 0.1392) |
|  |  |  |  |  |
| CDCP1 |  |  |  |  |
| Birth | 255 | -0.008 (-0.0500, 0.0274) | 245 | 0.0291 (-0.0115, 0.0911) |
| 1 day-3 months | 251 | -0.0053 (-0.0512, 0.0351) | 247 | 0.0319 (-0.0040, 0.0874) |
| >3-6 months | 255 | -0.0196 (-0.0779, 0.0243) | 255 | 0.0584 (0.0056, 0.1341) |
| >6-9 months | 255 | 0.0111 (-0.032, 0.0643) | 254 | 0.0154 (-0.0227, 0.0643) |
| >9-12 months | 255 | 0.0071 (-0.0321, 0.0496) | 255 | 0.0316 (-0.0115, 0.0965) |
|  |  |  |  |  |
| DNER |  |  |  |  |
| Birth | 255 | -0.0233 (-0.0734, 0.0107) | 245 | -0.0386 (-0.1157, 0.0088) |
| 1 day-3 months | 251 | -0.0077 (-0.0532, 0.0312) | 247 | -0.0200 (-0.0668, 0.0095) |
| >3-6 months | 255 | -0.0202 (-0.0785, 0.0234) | 255 | -0.023 (-0.0805, 0.0173) |
| >6-9 months | 255 | -0.0458 (-0.1128, -0.0026) | 254 | 0.0024 (-0.0404, 0.0461) |
| >9-12 months | 255 | -0.0277 (-0.0798, 0.0054) | 255 | -0.0177 (-0.0699, 0.0215) |
|  |  |  |  |  |
| IL18 |  |  |  |  |
| Birth | 255 | -0.0095 (-0.0678, 0.0465) | 245 | 0.0046 (-0.0630, 0.0784) |
| 1 day-3 months | 251 | -0.0199 (-0.0822, 0.0362) | 247 | 0.0237 (-0.0248, 0.0838) |
| >3-6 months | 255 | -0.0374 (-0.1161, 0.0281) | 255 | 0.0890 (0.0234, 0.1738) |
| >6-9 months | 255 | 0.0139 (-0.0470, 0.0840) | 254 | 0.0393 (-0.0126, 0.1082) |
| >9-12 months | 255 | 0.0219 (-0.0291, 0.0792) | 255 | 0.0408 (-0.0076, 0.1038) |
|  |  |  |  |  |
| OPG |  |  |  |  |
| Birth | 255 | 0.0384 (-0.0049, 0.1003) | 245 | 0.0235 (-0.0239, 0.0839) |
| 1 day-3 months | 251 | 0.0543 (0.0057, 0.1243) | 247 | -0.0077 (-0.0583, 0.0418) |
| >3-6 months | 255 | 0.0419 (-0.0025, 0.106) | 255 | -0.0044 (-0.0573, 0.0497) |
| >6-9 months | 255 | 0.0408 (-0.0026, 0.1041) | 254 | -0.0023 (-0.0559, 0.0538) |
| >9-12 months | 255 | 0.0196 (-0.0228, 0.0747) | 255 | 0.0068 (-0.0395, 0.0594) |
|  |  |  |  |  |
| SCF |  |  |  |  |
| Birth | 255 | -0.0706 (-0.1404, -0.0161) | 245 | 0.0592 (-0.0082, 0.1535) |
| 1 day-3 months | 251 | -0.0071 (-0.0587, 0.0435) | 247 | 0.0166 (-0.0342, 0.0787) |
| >3-6 months | 255 | -0.0022 (-0.0658, 0.0646) | 255 | -0.0043 (-0.0696, 0.0641) |
| >6-9 months | 255 | -0.0192 (-0.0874, 0.0463) | 254 | -0.0023 (-0.0660, 0.0652) |
| >9-12 months | 255 | -0.0152 (-0.0689, 0.0361) | 255 | -0.0063 (-0.0699, 0.0581) |

Mediated effects were adjusted for infant sex, primigravida, maternal age, maternal baseline parasitaemia, gestational age at enrolment, maternal education, and household wealth. Analysis includes all gravidae. The reference group was SP.

Inflammation protein names can be found in Table S1.

**Supplement 1. List of deviations from pre-analysis plan**

The analysis plan for this study was pre-specified at https://osf.io/f8wy4/. We note the following deviations from the plan:

1. We intended to employ principal components analysis and established pathway analysis and term enrichment databases, such as Blood Transcriptional Modules, Gene Ontology, and KEGG, to reduce the dimensionality and identify clusters of Olink inflammation-related biomarkers. However, these approaches did not yield discernible clusters. Therefore, we adhered to parametric regression, t-tests, volcano plots, and forest plots to investigate individual biomarkers. These approaches allowed us to systematically identify and track individual biomarkers demonstrating evidence of associations with the interventions and child growth outcomes for subsequent inclusion in our mediation models.

The 2D PCA plot shows the distribution of the observations along the first two principal components, with each point representing an individual observation coloured according to the intervention group. The ellipses represent the 95% confidence intervals for each group. Upon examination, the points corresponding to the SP and DP groups are intermixed, and the ellipses exhibit considerable overlap. This indicates that there is no strong separation between the treatment groups along the first two principal components. Consequently, the primary sources of variation captured by these components do not distinctly differentiate between the DP and SP groups.

1. The hypothesised causal pathways of the study as depicted in Figure S1 were sequential. However, due to limitations in available R programming tools, we decided to focus on single-mediator models to examine each intervention-mediator-outcome combination separately.
2. We did not include growth velocity results in the main text due to limited space. However, they are included in Supplement 2.
